# Proinsulin-Reactive CD4 T Cells in the Islets of Type 1 Diabetes Organ Donors

**DOI:** 10.3389/fendo.2021.622647

**Published:** 2021-03-25

**Authors:** Laurie G. Landry, Amanda M. Anderson, Holger A. Russ, Liping Yu, Sally C. Kent, Mark A. Atkinson, Clayton E. Mathews, Aaron W. Michels, Maki Nakayama

**Affiliations:** ^1^ Barbara Davis Center for Childhood Diabetes, University of Colorado School of Medicine, Aurora, CO, United States; ^2^ Department of Pediatrics, University of Colorado School of Medicine, Aurora, CO, United States; ^3^ Diabetes Center of Excellence, Department of Medicine, Division of Diabetes, University of Massachusetts Medical School, Worcester, MA, United States; ^4^ Department of Pathology, Immunology, and Laboratory Medicine, College of Medicine, University of Florida, Gainesville, FL, United States; ^5^ Department of Immunology and Microbiology, University of Colorado School of Medicine, Aurora, CO, United States

**Keywords:** antigens, type 1 diabetes, islets, T cell receptors, preproinsulin, epitopes

## Abstract

Proinsulin is an abundant protein that is selectively expressed by pancreatic beta cells and has been a focus for development of antigen-specific immunotherapies for type 1 diabetes (T1D). In this study, we sought to comprehensively evaluate reactivity to preproinsulin by CD4 T cells originally isolated from pancreatic islets of organ donors having T1D. We analyzed 187 T cell receptor (TCR) clonotypes expressed by CD4 T cells obtained from six T1D donors and determined their response to 99 truncated preproinsulin peptide pools, in the presence of autologous B cells. We identified 14 TCR clonotypes from four out of the six donors that responded to preproinsulin peptides. Epitopes were found across all of proinsulin (insulin B-chain, C-peptide, and A-chain) including four hot spot regions containing peptides commonly targeted by TCR clonotypes derived from multiple T1D donors. Of importance, these hot spots overlap with peptide regions to which CD4 T cell responses have previously been detected in the peripheral blood of T1D patients. The 14 TCR clonotypes recognized proinsulin peptides presented by various HLA class II molecules, but there was a trend for dominant restriction with HLA-DQ, especially T1D risk alleles DQ8, DQ2, and DQ8-trans. The characteristics of the tri-molecular complex including proinsulin peptide, HLA-DQ molecule, and TCR derived from CD4 T cells in islets, provides an essential basis for developing antigen-specific biomarkers as well as immunotherapies.

## Introduction

Type 1 diabetes (T1D) is a disease resulting from dysregulation of adaptive immune responses targeting pancreatic beta cells (1, 2). A high proportion of T1D patients have HLA-DR4-DQ8 and/or DR3-DQ2 haplotypes (3–7). This strong genetic association within the HLA class II gene locus suggests a crucial role of CD4 T cells in the development of this disease. Recent studies have demonstrated that CD4 T cells reactive to beta cell antigens are present in the islets of organ donors having T1D (8–11). Some of these T cells target peptides derived from proinsulin, providing an underlying rationale to develop proinsulin-specific immunotherapies. A number of antigen-specific immunotherapies aiming to modulate T1D-associated T cells, i.e., nanoparticle vaccination, small molecules or monoclonal antibodies to block formation of the tri-molecular complex, and infusion of antigen-specific regulatory T cells, require precise molecular information about peptide-MHC complexes targeted by T cells (12–19). Hence, identification of not only epitopes but in addition, the MHC molecules presenting these peptides, will aid in the development of antigen-specific immunotherapies targeting T cells associated with the T1D pathogenesis.

Preproinsulin is selectively and abundantly expressed by pancreatic beta cells, thereby often being highlighted as a key autoantigen for T1D. Autoantibodies directed to insulin and proinsulin are observed in sera from a majority of individuals developing T1D (20, 21). As evidence of antigens for T cells, Mallone and colleagues reported that approximately one third of peptides eluted from HLA class I molecules expressed by primary human islets were derived from preproinsulin (22). Furthermore, we and others have isolated T cells specific to preproinsulin in the islets and peripheral blood of individuals having T1D (8–10, 23–27). In particular, Mannering and colleagues identified CD4 T cell clones reactive to C-peptide, all of which recognized the peptide presented by HLA-DQ and/or DQ8-trans, in an islet sample from an organ donor having T1D (8). They subsequently demonstrated T cell responses to C-peptide in the peripheral blood of T1D patients and consistent with their islet study, the majority of C-peptide-reactive T cell clones isolated from peripheral blood samples were restricted with HLA-DQ molecules as well (27). Kent and colleagues also isolated preproinsulin-reactive T cell lines from islet samples of multiple T1D organ donors (9). In our previous study, we identified T cell receptor (TCR) clonotypes expressed by T cells in the islets of T1D organ donors and found three clonotypes specific to insulin B-chain and C-peptide presented by HLA-DQ8 and DQ8-trans (10). Thus, a number of studies have demonstrated the presence of preproinsulin-reactive T cells in the islets of individuals having T1D. In this study, we aimed to comprehensively analyze and characterize tri-molecular complexes composed of preproinsulin peptide, HLA, and TCR derived from CD4 T cells in the islets of T1D organ donors.

Recent improvements in sequencing technologies have facilitated the identification of T and B cell receptor sequences in a high-throughput manner (28, 29). It is desirable to determine antigen specificity of these immunoreceptors and indeed, there has been remarkable progress in developing high-throughput strategies to identify antigens recognized by these antigen receptors (30–37). Unlike analysis of B cell antigens, the fact that MHC molecules participate in the recognition of epitopes by TCRs makes it more complicated to identify T cell targets. Immortalized cell lines expressing TCR clonotypes of interest promoted feasibility of directly evaluating reactivity to specific peptide-MHC complexes. Hence, we recently developed a multiplex assay system that can simultaneously assess reactivity of a number of TCR transductant cell lines (38). Multiplex efforts spare samples and reagents necessary for the assay, thereby allowing analysis of reactivity to hundreds of peptides within a relatively short period of time. In this current study, we used this multiplex assay system to test responses against preproinsulin peptides in the presence of autologous B cells transformed with Epstein-Barr virus (EBV) by islet-derived TCR clonotypes obtained from the residual islets of T1D organ donors. The peptide library is composed of 12-15mers of peptides that are generated from the natural form of preproinsulin. Thus, we evaluated reactivity to preproinsulin epitopes presented by all possible HLA class II molecules in a deep and comprehensive manner. Furthermore, using antigen presenting cell lines that exclusively express a single HLA, we identified HLA class II molecules presenting peptides to individual preproinsulin-reactive TCR clonotypes. Here we report the molecular elements involved in the immune-recognition of preproinsulin peptides by islet-derived CD4 T cells.

## Results and Discussion

### Screening of Islet CD4 T Cell-Derived T Cell Receptors for the Response to Preproinsulin Peptides

**Figure 1:**
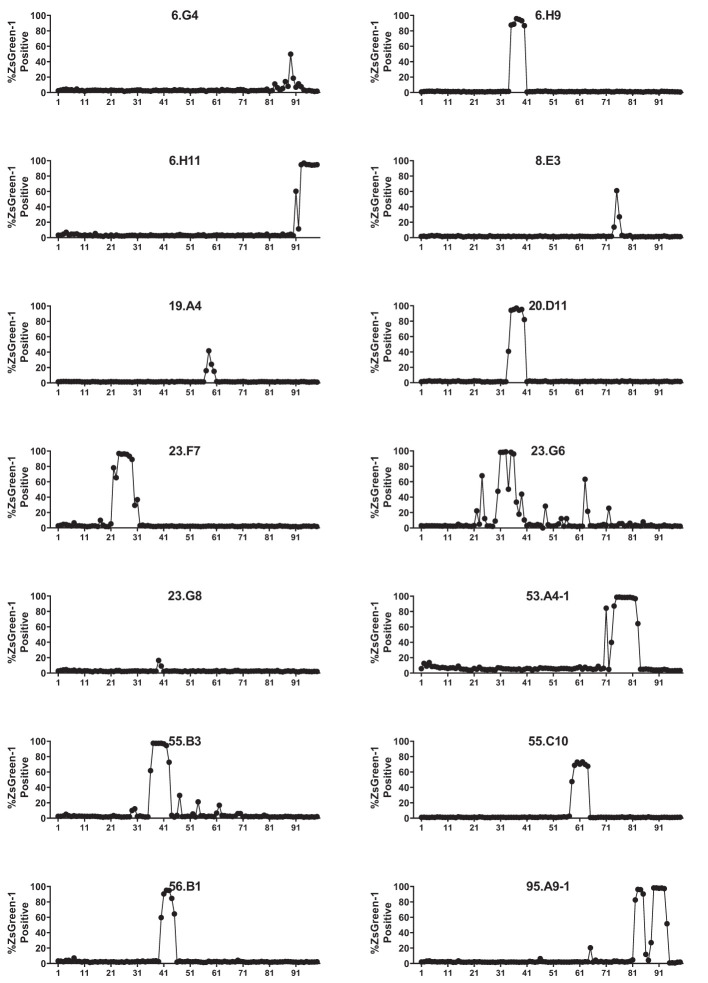
Screening for the response to a preproinsulin truncated peptide library. 5KC T-hybridoma cells expressing ZsGreen-1 upon activation were used to express each TCR clonotype. TCR transductants were cultured with 99 truncated peptide pools containing 12-, 13-, 14-, and 15-mers of peptides ending at the same position of preproinsulin in the presence of autologous EBV-transformed B cells. After overnight culture, cells were evaluated for ZsGreen-1 expression by flow cytometry. Percentages of ZsGreen-1-positive cells in response to each peptide pool are shown for 14 TCR transductants that responded to one or more peptide pools.

**Figure 2:**
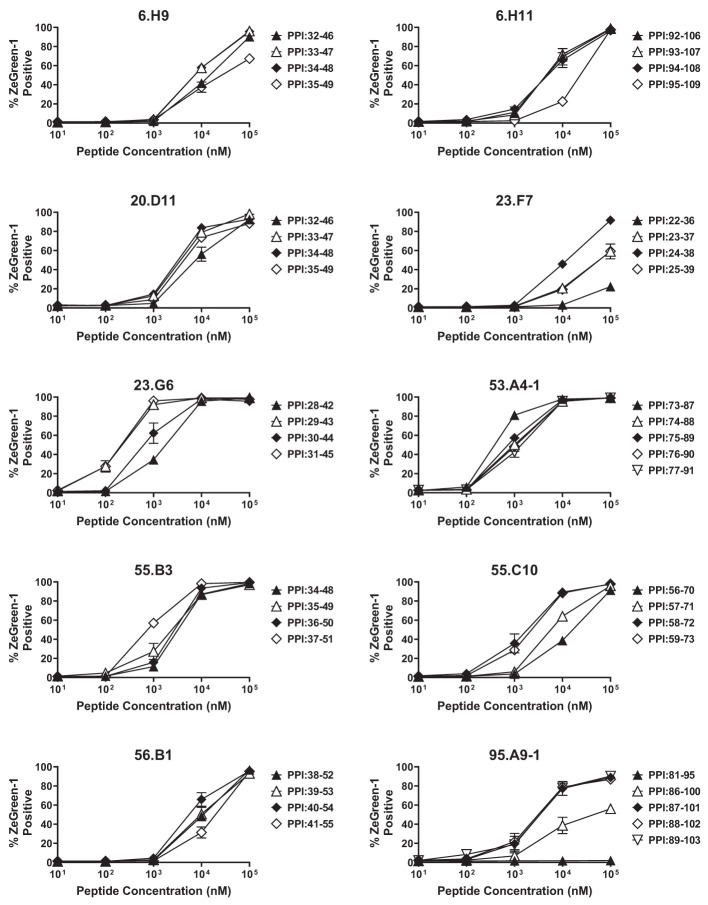
Determining optimal peptides. TCR transductants that responded to multiple peptide pools in the screening were tested for the response to newly synthesized 15-mer peptides contained in individual peptide pools in the presence of autologous EBV-transformed B cells, and were evaluated for ZsGreen-1 expression by flow cytometry. Experiments were independently repeated three times, and mean values ± standard error of the mean are shown.

We analyzed 187 TCR clonotypes derived from 166 clonal CD4 T cells in the islets of six T1D organ donors for specificity to preproinsulin (**Table 1**, **Supplementary Table 1**). These TCR clonotypes were selected for analysis because they were detected from multiple cells or have a specific V-gene motif such as TRAV13-1, TRAV26-1, or TRAV38-2, which are preferentially used by CD4 T cells specific to amino acids 9-23 of insulin B chain (10, 26, 39). We expressed each TCR clonotype in a 5KC murine T-hybridoma cell line, which has an added activation reporter driven by the production of nuclear factor of activated T cells (NFAT) (38), to test the response to 99 peptide pools containing 12-mer to 15-mer of peptides derived from preproinsulin (**Supplementary Table 2**). To detect responses against peptides presented by any possible HLA molecules expressed by a given donor, autologous EBV-transformed B cells were used. Screening of 187 TCR clonotypes identified 14 TCRs (7.5%), derived from four donors, that responded to preproinsulin peptide pools. These 14 TCR transductants expressed a fluorescent reporter, ZsGreen-1, when cultured with particular peptide pools (**Figure 1**). As the peptide concentration used for the screening was supraphysiologic to maximize the detection of preproinsulin-reactive TCRs, some TCR transductants responded to multiple peptide pools that contain peptides sharing the same portion of amino acid sequences. To determine an optimal epitope region, we newly synthesized 15-mer peptides contained in top 4 or 5 peptide pools that most efficiently stimulated TCR transductants (**Supplementary Figure 1**, **Supplementary Table 3**) and used those peptides to evaluate responses by each TCR transductant. TCR transductants reacted with peptide pools in a dose-dependent manner (**Figure 2**), and thus we identified the region of peptide containing the optimal epitope for each TCR clonotype. Overall, we found seven, four, and three epitopes recognized by the 14 TCR clonotypes in insulin B-chain, C-peptide, and A-chain, respectively, including three TCRs, 6.H9, 20.D11, and 8.E3, that were previously found by screening of the response to overlapping preproinsulin peptides (10). Altogether, we identified 14 preproinsulin-reactive TCR clonotypes in the islets of four out of six T1D organ donors studied.

**Table 1:** HLA class II alleles of T1D organ donors and the numbers of T cells analyzed.

Donor ID	HLA-DRB1*	DQA1-DQB1*	DPA1-DPB1*	Number of clonal T cells analyzed	Number of unique TCRs analyzed	Number of PPI-reactive TCRs
nPOD 69	04:01 (DR4) 07:01 (DR7)	03:01-03:02 (DQ8) 02:01-02:02 (DQ2.2)	01:03-03:01 01:03-04:01 (DP4)	7	7	0
nPOD 6323	03:01 (DR17 [DR3]) 04:02 (DR4)	05:01-02:01 (DQ2.5) 03:01-03:02 (DQ8)	01:03-04:01 (DP4)	52	56	4
nPOD 6342	04:01 (DR4) 01:01 (DR1)	03:01-03:02 (DQ8) 01:01-05:01 (DQ5)	01:03-04:01 (DP4)	34	40	5
nPOD 6414	03:01 (DR17 [DR3]) 09:01 (DR9)	05:01-02:01 (DQ2.5) 03:03-02:02 (DQ2.3)	01:03-04:01 (DP4) 01:04-15:01	34	40	4
nPOD 6472	03:01 (DR17 [DR3]) 04:04 (DR4)	05:01-02:01 (DQ2.5) 03:01-03:02 (DQ8)	01:03-04:01 (DP4) 02:01-10:01	30	33	1
nPOD 6367	04:01 (DR4) 07:01 (DR7)	03:01-03:02 (DQ8) 02:01-02:02 (DQ2.2)	01:03-02:01 06:01-11:01	9	11	0

*Nomenclatures in parentheses indicate HLA serotypes.

### Identification of Peptide-MHC Complexes Targeted by Proinsulin-Reactive T Cell Receptor Clonotypes

**Figure 3:**
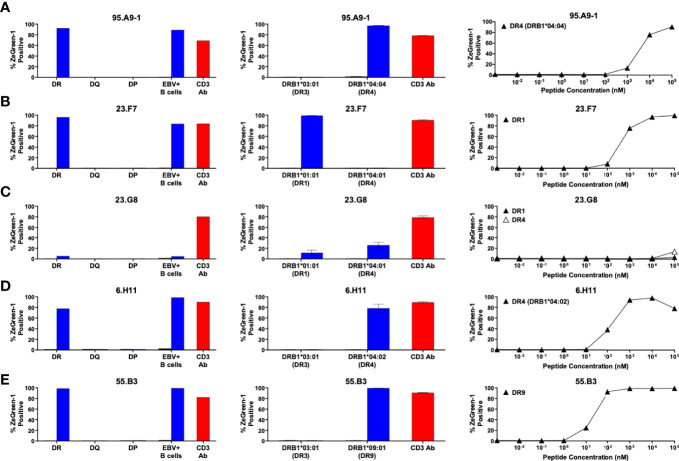
Preproinsulin-reactive TCR clonotypes restricted by HLA-DR. TCR transductants, **(A)** 95.A9-1, **(B)** 23.F7, **(C)** 23.G8, **(D)** 6.H11, **(E)** 55.B3, were cultured with cognate peptides in the presence of designated antigen presenting cells, followed by assessment of ZsGreen-1 expression by flow cytometry. Left panels: TCR transductants were cultured with (blue bars) or without (black bars) cognate peptides in the presence of autologous EBV-transformed B cells generated from spleen cells of **(A)** nPOD 6472, **(B, C)** nPOD 6342, **(D)** nPOD 6323, or **(E)** nPOD 6414, or K562 cells transduced with DR, DQ, or DP alleles based upon their HLA genotype in **Table 1**. Culture wells containing an anti-CD3 monoclonal antibody were included as a positive control (red bars). Center panels: TCR transductants were cultured with (blue bars) or without (black bars) cognate peptides in the presence of K562 cells transduced with individual DR alleles based upon their HLA genotype in **Table 1**. Right panels: TCR transductants were cultured with different concentrations of cognate peptides in the presence of K562 cells transduced with **(A)** DRA1*01:01 and DRB1*04:04; **(B)** DRA1*01:01 and DRB1*01:01; **(C)** DRA1*01:01 and DRB1*01:01 (black triangles), or DRA1*01:01 and DRB1*04:01 (white triangles); **(D)** DRA1*01:01 and DRB1*04:02; **(E)** DRA1*01:01 and DRB1*09:01. Experiments in left panels were performed once. All remaining experiments in center and right panels were independently repeated three times, and mean values ± standard error of the mean are shown.

We next aimed to identify HLA molecules presenting proinsulin peptides to the 14 TCR clonotypes. We used the newly synthesized 15-mer peptides that were identified as a region containing an optimal epitope for each TCR transductant. Our strategy was to test the response to the cognate peptide in the presence of K562 antigen presenting cells expressing HLA molecules identical to those of the TCR donor. K562 cells do not express endogenous HLA class II molecules, allowing us to examine the response restricted only by the introduced HLA. We first generated three K562 cell lines expressing HLA-DR (DRB1), DQ, or DP molecules matching each donor and used them as antigen presenting cells. Once narrowing the presenting HLA molecules to DR, DQ, or DP-derived molecules, we further examined the responses by TCR transductants in the presence of K562 cell lines that individually express any possible combination of HLA-DR, DQ, or DP molecules including cis- and trans-combinations of HLA-DQA1 and DQB1 or DPA1 and DPB1 molecules. This way, we identified one or more HLA molecules presenting the cognate peptide to each TCR. For example, the 95.A9-1 TCR clonotype was derived from donor nPOD 6472, who had the DR3 and DR4 haplotypes (**Table 1**). The 95.A9-1 transductants responded to a cognate peptide, preproinsulin 87-101, when cultured with K562 cells co-transduced with DRA1*01:01, DRB1*04:04, and DRB1*03:01, but not with those transduced with the DQ or DP alleles (**Figure 3A**, left panel). The 95.A9-1 TCR transductant cells were further examined for the response to peptides in the presence of K562 cells transduced with DRA1*01:01 along with either DRB1*04:04 or DRB1*03:01 and were activated only when co-cultured with cells expressing DRB1*04:04 (**Figure 3A**, center panel). Lastly, we confirmed that the 95.A9-1 cells responded to the cognate peptide in the presence of K562 cells expressing the determined HLA in a dose-dependent manner (**Figure 3A**, right panel). Thus, we determined that 95.A9-1 is reactive to preproinsulin 87-101 presented by DRA1*01:01-DRB1*04:04 (DR4).

**Figure 4:**
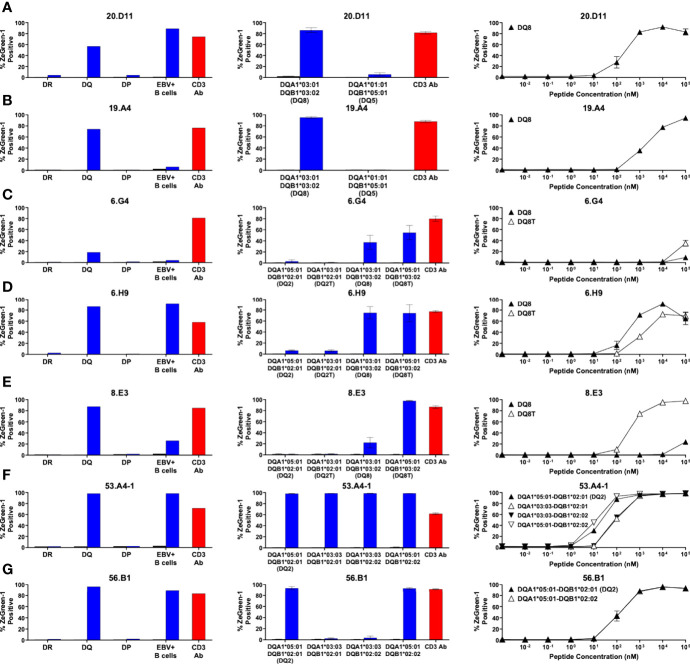
Preproinsulin-reactive TCR clonotypes restricted by HLA-DQ. TCR transductants, **(A)** 20.D11, **(B)** 19.A4, **(C)** 6.G4, **(D)** 6.H9, **(E)** 8.E3, **(F)** 53.A4-1, **(G)** 56.B1, were cultured with cognate peptides in the presence of designated antigen presenting cells, followed by assessment of ZsGreen-1 expression by flow cytometry. Left panels: TCR transductants were cultured with (blue bars) or without (black bars) cognate peptides in the presence of autologous EBV-transformed B cells generated from spleen cells of **(A, B)** nPOD 6342, **(C–E)** nPOD 6323, or **(F, G)** nPOD 6414, or K562 cells transduced with DR, DQ, or DP alleles based upon their HLA genotype in **Table 1**. Culture wells containing an anti-CD3 monoclonal antibody were included as a positive control (red bars). Center panels: TCR transductants were cultured with (blue bars) or without (black bars) cognate peptides in the presence of K562 cells transduced with individual DQ alleles based upon their HLA genotype in **Table 1**. Right panels: TCR transductants were cultured with different concentrations of cognate peptides in the presence of K562 cells transduced with **(A, B)** DQA1*03:01 and DQB1*03:02; **(C–E)** DQA1*03:01 and DQB1*03:02 (black triangles), or DQA1*05:01 and DQB1*03:02 (white triangles); **(F)** DQA1*05:01 and DQB1*02:01 (black triangles), DQA1*03:03 and DQB1*02:01 (white triangles), DQA1*03:03 and DQB1*02:02 (black inverted triangles), or DQA1*05:01 and DQB1*02:02 (white inverted triangles); **(G)** DQA1*05:01 and DQB1*02:01 (black triangles), or DQA1*05:01 and DQB1*02:02 (white triangles). Experiments in left panels were performed once. All remaining experiments in center and right panels were independently repeated three times, and mean values ± standard error of the mean are shown.

**Figure 5:**
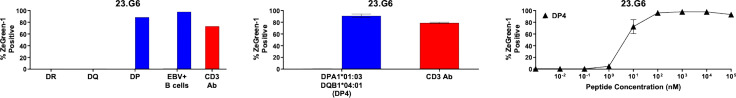
Preproinsulin-reactive TCR clonotypes restricted by HLA-DP. The TCR 23.G6 transductant was cultured with cognate peptides in the presence of designated antigen presenting cells, followed by assessment of ZsGreen-1 expression by flow cytometry. Left panel: The 23.G6 TCR transductants were cultured with (blue bars) or without (black bars) cognate peptides in the presence of autologous EBV-transformed B cells generated from spleen cells of nPOD 6342, or K562 cells transduced with DR (DRA1*01:01, DRB1*04:01, DRB1*01:01), DQ (DQA1*03:01, DQA1*01:01, DQB1*03:02, DQB1*05:01), or DP (DPA1*01:03, DPB1*04:01) alleles. Culture wells containing an anti-CD3 monoclonal antibody were included as a positive control (red bars). Center panel: The 23.G6 TCR transductant was cultured with (blue bars) or without (black bars) cognate peptides in the presence of K562 cells transduced with a DP gene combination, DPA1*01:03 and DPB1*04:01. Right panel: The 23.G6 TCR transductant was cultured with different concentrations of cognate peptides in the presence of K562 cells transduced with DPA1*01:03 and DPB1*04:01. The experiment in the left panel was performed once. Experiments in the center and right panels were independently repeated three times, and mean values ± standard error of the mean are shown.

Using the same strategy, we determined HLA molecules presenting a cognate peptide to all individual TCR clonotypes except one, 55.C10. **Figures 3**–**5** show the results (left panels: determining HLA-DR, DQ, or DP, center panels: determining HLA-alpha and -beta combinations, right panels: dose-response assessment) for five DR- (**Figure 3**), seven DQ- (**Figure 4**), and one DP (**Figure 5**)-restricted TCRs.

**Figure 6:**
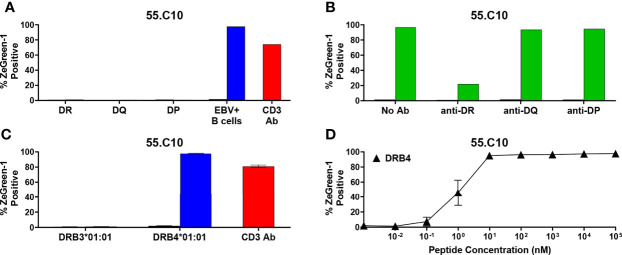
Preproinsulin-reactive TCR clonotypes restricted by HLA-DRB4. **(A)** The 55.C10 TCR transductant was cultured with (blue bars) or without (black bars) cognate peptides in the presence of autologous EBV-transformed B cells generated from spleen cells of nPOD 6414, or K562 cells transduced with DR (DRA1*01:01, DRB1*03:01, DRB1*09:01), DQ (DQA1*05:01, DQA1*03:03, DQB1*02:01, DQB1*02:02), or DP (DPA1*01:03, DPA1*01:04, DPB1*04:01, DPB1*15:01) alleles. Culture wells containing an anti-CD3 monoclonal antibody were included as a positive control (red bars). **(B)** The 55.C10 TCR transductant was cultured with (green bars) or without (black bars) cognate peptides in the presence or absence of anti-DR, -DQ, or -DP antibodies. Autologous nPOD 6414 EBV-transformed B cells were used as antigen presenting cells. **(C)** The 55.C10 TCR transductant was cultured with (blue bars) or without (black bars) cognate peptides in the presence of K562 cells transduced with DRB3 (DRA1*01:01 and DRB3*01:01) or DRB4 (DRA1*01:01 and DRB4*01:01) alleles. **(D)** The 55.C10 TCR transductant was cultured with different concentrations of cognate peptides in the presence of K562 cells transduced with DRA1*01:01 and DRB4*01:01. Experiment in panels **(A, B)** were performed once. All remaining experiments in panels **(C, D)** were independently repeated three times, and mean values ± standard error of the mean are shown.

There was one TCR clonotype, 55.C10, that did not respond to a cognate peptide in the presence of any K562 cell lines expressing the donor’s HLA-DR, DQ, and DP molecules (**Figure 6A**). To investigate which HLA genes restrict 55.C10, we tested the response by TCR transductants in the presence of autologous B cells and antibodies against HLA-DR, DQ, or DP and found that anti-HLA-DR antibodies suppressed the response (**Figure 6B**). The TCR is likely to recognize the peptide presented by HLA-DR, but K562 cells expressing molecules derived from the DRB1 allele was not able to stimulate the TCR transductant, suggesting that another DR beta chain such as those derived from the DRB3, DRB4, or DRB5 genes may be involved in the presentation of the peptide. The donor of TCR 55.C10, nPOD 6414, had DR3 and DR9 haplotypes, which are linked to functional DRB3 and DRB4 genes, respectively, but DRB5 alleles of both haplotypes are pseudogenes. After determining alleles of DRB3 and DRB4 of the donor (i.e., DRB3*01:01 and DRB4*01:01), we generated K562 cells transduced with these allelic variants and found that the 55.C10 TCR transductants were reactive to the peptide presented by HLA-DRB4*01:01 (**Figures 6C, D**). As CD4 T cells specific to another proinsulin peptide presented by DRB4*01:01 have been observed in the blood of T1D patients (40), there may be disease-specific epitopes presented by HLA-DRB3 and DRB4 alleles that are linked to the T1D-susceptible HLA-DR4-DQ8 and/or DR3-DQ2 haplotypes.

**Figure 7:**
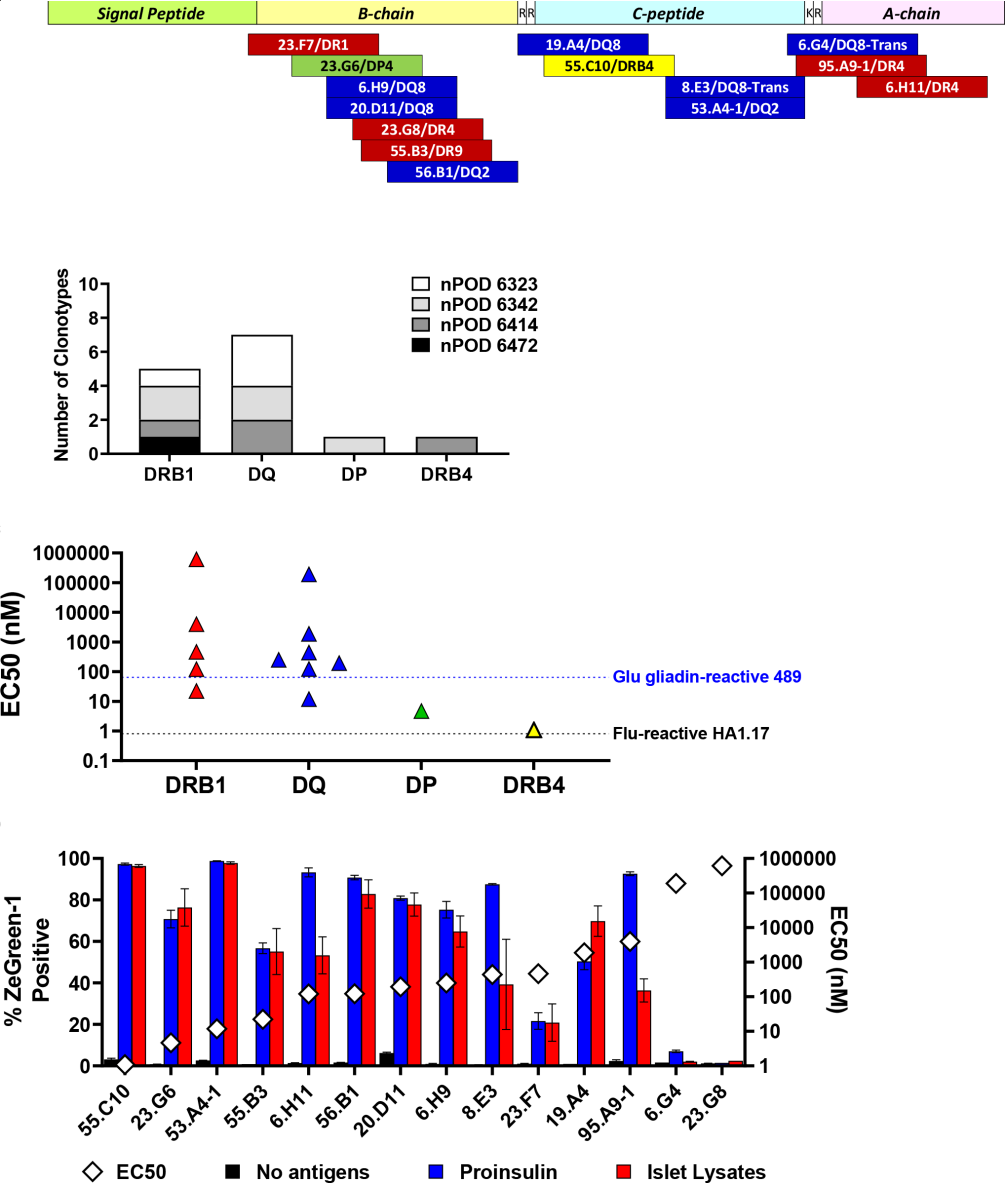
Characteristics of proinsulin-specific TCR clonotypes. **(A)** Location of epitopes in preproinsulin and restricting HLA molecules are mapped. Red, blue, green, and yellow bars represent epitopes for TCR clonotypes restricted by HLA-DRB1, DQ, DP, and DRB4. **(B)** Numbers of preproinsulin-reactive TCR clonotypes restricted by HLA-DRB1, DQ, DP, and DRB4. Preproinsulin-reactive TCR clonotypes derived from individual nPOD donors are shown separately by HLA restriction. **(C)** EC_50_ values of responses by preproinsulin-reactive TCR clonotypes are shown separately by HLA restriction. EC_50_ values of responses by HA.1.17 to the cognate influenza peptide (black line) and 489 to the deamidated alpha-gliadin peptide (blue line) are included for reference. **(D)** Responses to proinsulin and human islet lysates by TCR transductants reactive to proinsulin peptides. TCR transductants were cultured with K562 cells expressing cognate HLA molecules pulsed with proinsulin (blue bars) or islet lysates (red bars), or without antigens (black bars). After overnight culture, ZsGreen-1 expression was evaluated by flow cytometry. Experiments were independently repeated twice, and mean values ± standard error of the mean are shown. EC_50_ values of responses to cognate peptides (white diamond symbols) are included for reference.

**Table 2** as well as **Figure 7A** summarize peptides and presenting HLA molecules targeted by individual TCR clonotypes. Of note, peptides recognized by multiple TCRs, such as preproinsulin 33-47 recognized by 6.H9 and 20.D11, preproinsulin 36-50 recognized by 23.G8 and 55.B3, and preproinsulin 72-87 recognized by 8.E3 and 53.A4-1, were presented by the same HLA gene products (i.e., DR or DQ) but sometimes different allele products. In addition, it was noted that several TCR clonotypes, 23.G8, 6.H9, 8.E3, 53.A4-1, and 6.G4, recognized cognate peptides presented by several HLA class II molecules, albeit with different levels of response, implying that the same peptides can be presented by multiple HLA molecules and induce various levels of T cell activation. These different levels of responsiveness are likely to be the combined outcome from differential peptide to HLA binding affinity, and TCR to peptide-MHC complex affinity. Intensity of signals provided through the TCR engagement is a major factor determining T cell phenotype as well as their fate (41, 42). It will be important to elucidate how T cells determine their responses when receiving different levels of TCR signaling through epitopes presented by multiple HLA class II molecules having different affinities.

Overall, we identified five DRB1, seven DQ, one DP, and one DRB4-restricted TCR clonotypes that recognize proinsulin peptides (**Figure 7B**). It has been suggested that DRB1-restricted TCR repertoires are generally the largest among those expressed by CD4 T cells. While the number of proinsulin-specific clonotypes identified in our study is small, there was a noteworthy trend that the proportion of DR-restricted TCR clonotypes was not as high as previously reported frequencies of DR-restricted TCRs in virus-reactive repertoires (43–45), and rather DQ-restricted TCRs were more predominant. Future studies investigating HLA restriction of a large number of T cells from the islets of patients and at-risk individuals are expected to confirm this trend.

### Responsiveness to Proinsulin Epitopes by Autoreactive T Cells

The responsiveness to peptides differed by individual TCRs (**Figures 3**–**5**, right panels). To compare response levels of preproinsulin-reactive TCRs with those of TCRs unrelated to the T1D pathogenesis, we generated two TCR transductant cell lines using the same method to make islet TCR transductants. First, we used an influenza virus-reactive TCR clonotype, HA1.17. The HA1.17 TCR transductants reacted to a cognate influenza peptide presented by either HLA-DR1 or DR4 starting at only one nanomolar (**Supplementary Figure 2A**). Next, we evaluated the sensitivity of a gliadin-specific TCR, 489, to the native and deamidated forms of peptides in our transductant system. It is well-appreciated that alpha-gliadin peptides that undergo deamidation (Q→E) are target antigens for T cells involved in the pathogenesis of celiac disease, an autoimmune disease caused by gluten intake in disease-sensitive individuals (46, 47). As expected, the 489 TCR transductants responded to the deamidated peptide presented by DQ8 more sensitively than the native alpha-gliadin peptide (**Supplementary Figure 2B**). Several proinsulin-reactive TCR clonotypes including two restricted by HLA-DP and DRB4 showed comparable levels of responses to their cognate peptides compared to those of the influenza-specific HA1.17 and the gliadin-specific 489 TCRs (**Figure 7C**). The expression levels of HLA-DP and DRB4 molecules on antigen presenting cells are reported to be lower than that of HLA-DRB1 molecules (45, 48), which may influence selection and differentiation of self-reactive T cells restricted by HLA molecules with lower cell surface expression, such as HLA-DP. On the other hand, responsiveness of several preproinsulin-specific clonotypes was lower than those of the HA1.17 and 489 TCRs (**Figure 7C**). In particular, the 6.G4 and 23.G8 TCRs needed excessive amounts of peptides to be activated. There is a possibility that biological targets for these TCR clonotypes exhibiting weaker responses could be neoepitopes such as those receiving post-translational modifications and products resulting from alternative splicing. Alternatively, antigens activating T cells expressing these TCRs could be unrelated to proinsulin or even other islet antigens, such as those derived from microbes, and the TCRs may be cross-reactive to proinsulin.

### Reactivity to Proinsulin and Islet Tissues

To validate tissue specificity, we further tested the response to whole proinsulin and tissue lysates made from primary human islets by the 14 TCR transductants reactive to proinsulin peptides. All except two TCRs, 6.G4 and 23.G8, which showed only marginal responses to their cognate peptides, responded to islet lysates (**Figure 7D**). Consistent with undetectable reactivity to islet lysates, 6.G4 but not 23.G8 weakly responded to proinsulin. These observations are compatible with the possibility of cross-reactivity to proinsulin. Intensities of responses to cognate peptides are not necessarily associated with those to whole proinsulin and islet lysates, and some TCRs such as 6.H11, 8.E3, and 95.A9-1 preferred to react with whole proinsulin rather than islet lysates. These discrepancies may be caused by difference between optimal epitopes and peptides formed islet cells or antigen presenting cells.

### Citrullinated Insulin B-Chain Peptides Do Not Enhance T Cell Responses

**Figure 8:**
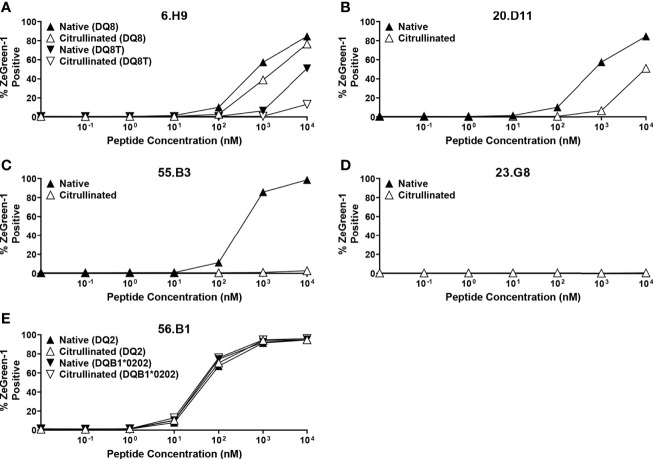
Responses to citrullinated and native insulin B-chain peptides. TCR transductants, **(A)** 6.H9, **(B)** 20.D11, **(C)** 55.B3, **(D)** 23.G8, **(E)** 56.B1, were cultured with different concentrations of insulin B-chain peptide (SHLVEALYLVCGE-**R**-GFFYTPK) (black symbols) or that with citrullination (SHLVEALYLVCGE-**Cit**-GFFYTPK) (white symbols) in the presence of K562 cells expressing the following HLA molecules for each TCR clonotype: **(A)** DQ8 (DQA1*03:01-DQB1*03:02) (triangles) and DQ8T (DQA1*05:01-DQB1*03:02) (inverted triangles); **(B)** DQ8 (DQA1*03:01-DQB1*03:02) (triangles); **(C)** DR9 (DRA1*01:01-DRB1*09:01) (triangles); **(D)** DR4 (DRA1*01:01-DRB1*04:01) (triangles); **(E)** DQ2 (DQA1*05:01-DQB1*02:01) (triangles) and (DQA1*05:01-DQB1*02:02) (inverted triangles). All experiments were independently repeated three times, and mean values ± standard error of the mean are shown.

Five out of 14 proinsulin-reactive TCR transductants responded to peptides located in the latter portion of insulin B-chain, and the majority of these TCRs showed relatively weak responsiveness to the native insulin peptides. Citrullination converts arginine to citrulline and is an important post-translational modification of self-antigens in autoimmune diseases such as Rheumatoid arthritis and T1D (49–51). To begin to examine the potential for neoepitopes within proinsulin, we investigated antigenicity of a citrullinated insulin B-chain peptide. We tested the five insulin B-chain-reactive TCR transductants for response to the citrullinated and the native forms of insulin B-chain peptides. All TCR transductants except 23.G8, that reacted with the cognate 15-mer peptide very weakly (**Figure 3C**), responded to the native insulin B-chain peptide, but citrullination of the peptide did not induce stronger responses in any TCRs (**Figure 8**). Further studies to identify biological epitopes, especially for TCRs with low responses to the natural form of proinsulin, such as 23.G8, will be important to understand the mechanisms of initiating and driving anti-islet autoimmunity.

In the current study, we identified 11 new proinsulin-reactive TCR clonotypes in addition to three clonotypes found in our previous study that screened reactivity to ten overlapping preproinsulin peptides (10), and thus 14 TCR clonotypes were characterized for preproinsulin specificity and HLA restriction. The use of a truncated peptide library containing 99 preproinsulin peptide pools and autologous EBV-transformed B cell lines facilitated our ability to identify these additional TCR clonotypes. Epitopes were found in B-chain, C-peptide, and A-chain regions, and there were regions that contained multiple epitopes recognized by TCRs derived from different donors (**Table 2**, **Figure 7A**). Some of these epitopes were identified as targets for islet-derived CD4 T cell clones observed in previous studies by others (8, 9). Overall, there are several peptide regions spanning about 20 amino acids, i.e. preproinsulin 33-53 (insulin B-chain), preproinsulin 55-71 (C-peptide), preproinsulin 72-90 (C-peptide), preproinsulin 86-101 (A-chain), that are commonly targeted by CD4 T cells in the islets of individuals having T1D. Inflammatory responses by CD4 T cells to these “hot spot” regions have previously been observed in peripheral blood samples of T1D patients (26, 27, 52–55). While it is unknown whether CD4 T cells reactive to the preproinsulin hot spots in the blood are clonally identical to those in the islets, it is noteworthy that the same antigen specificities have been confirmed in blood and islets.

**Table 2:** HLA class II alleles of T1D organ donors and the numbers of T cells analyzed.

nPOD Donor	TCR ID	Peptide	Peptide Sequence	Region in Proinsulin	HLA*#
6342	23.F7	PPI:24-38	AFVNQHLCGSHLVEA	B-chain	DRB1*01:01 (DR1)
6342	23.G6	PPI:29-43	HLCGSHLVEALYLVC	B-chain	DPA1*01:03-DPB1*04:01 (DP4)
6323	6.H9	PPI:33-47	SHLVEALYLVCGERG	B-chain	DQA1*03:01-DQB1*03:02 (DQ8) DQA1*05:01-DQB1*03:02 (DQ8-trans)
6342	20.D11	PPI:33-47	SHLVEALYLVCGERG	B-chain	DQA1*03:01-DQB1*03:02 (DQ8)
6342	23.G8	PPI:36-50	VEALYLVCGERGFFY	B-chain	DRB1*04:01 (DR4), DRB1*01:01 (DR1)
6414	55.B3	PPI:37-51	EALYLVCGERGFFYT	B-chain	DRB1*09:01 (DR9)
6414	56.B1	PPI:40-54	YLVCGERGFFYTPKT	B-chain	DQA1*05:01-DQB1*02:01 (DQ2.5) DQA1*05:01-DQB1*02:02
6342	19.A4	PPI:55-69	RREAEDLQVGQVELG	(RR) C-peptide	DQA1*03:01-DQB1*03:02 (DQ8)
6414	55.C10	PPI:58-72	AEDLQVGQVELGGGP	C-peptide	DRB4*01:01 (DR53)
6323	8.E3	PPI:72-87	PGAGSLQPLALEGSLQ	C-peptide	DQA1*05:01-DQB1*03:02 (DQ8-trans) DQA1*03:01-DQB1*03:02 (DQ8)
6414	53.A4-1	PPI:72-87	PGAGSLQPLALEGSLQ	C-peptide	DQA1*05:01-DQB1*02:01 (DQ2.5) DQA1*05:01-DQB1*02:02 DQA1*03:03-DQB1*02:02 (DQ2.3) DQA1*03:03-DQB1*02:01
6323	6.G4	PPI:86-100	LQKRGIVEQCCTSIC	(B-KR) A-chain	DQA1*05:01-DQB1*03:02 (DQ8-trans) DQA1*03:01-DQB1*03:02 (DQ8)
6472	95.A9-1	PPI:87-101	QKRGIVEQCCTSICS	(B-KR) A-chain	DRB1*04:04 (DR4)
6323	6.H11	PPI:94-108	QCCTSICSLYQLENY	A-chain	DRB1*04:02 (DR4)

*Nomenclatures in parentheses indicate HLA serotypes.

^#^HLA allele combinations recognized by the TCR most efficiently are shown in bold.

It will be important to determine binding motifs as well as affinity of the epitopes to the restricting HLA molecules as these elements are essential to determine responsiveness of T cells and to develop therapies targeting peptide-MHC complexes. To begin, we conducted a computational simulation analysis to predict core epitope sequences using the Immune Epitope Database (IEDB) MHC-II Binding Prediction online tool (http://tools.iedb.org/mhcii/), which allows us to use multiple *in silico* prediction models. A number of simulations predicted identical amino acid motifs as a core epitope for each proinsulin-reactive clonotype (**Supplementary Table 4**). Further efforts validating binding affinities and T cell reactivities to these predicted epitopes are desired in the future.

All HLA-DR, DQ, and DP molecules were used to present peptides to proinsulin-reactive TCR clonotypes. However, there was a trend of restriction with HLA-DQ, particularly the T1D risk alleles, DQ8, DQ2, or DQ8-trans (**Table 2**, **Figure 7A**). Mannering and colleagues also reported that this preferred restriction with risk DQ alleles was observed in C-peptide-specific CD4 T cells in the blood (27). Our study extends this finding to islet-derived TCRs specific to other regions of proinsulin. Future studies to determine antigen specificity outside of preproinsulin (e.g., glutamic acid decarboxylase, zinc transporter 8, islet antigen 2) will clarify whether the trend of DQ restriction is a general feature of TCR clonotypes expressed by T1D-associated CD4 T cells. Additionally, it is important to elucidate the mechanisms by which HLA-DQ is preferentially used to present epitopes to T cells reactive to proinsulin and potentially other islet antigens. Whether this is a global phenomenon across patients or patient-specific will help design and personalize immune therapies to preserve endogenous beta-cell function in T1D. Notably, there is a therapy (methyldopa) being tested that specifically blocks self-antigen presentation by DQ8 and subsequent autoreactive T cell responses (12). Our results also indicate that antigen specific immunotherapies with insulin, should give strong consideration to including A-chain, B-chain, and C-peptide (e.g., all of proinsulin) as there are islet-derived CD4 T cell epitopes within all of these regions.

In conclusion, we identified 14 proinsulin-specific TCR clonotypes expressed by CD4 T cells in the islets of four out of six organ donors having T1D. These TCRs were restricted by various HLA class II molecules, but there was a trend of using T1D-risk conferring HLA-DQ molecules. There are four hot spots within proinsulin that contain epitopes preferentially targeted by the responding islet TCRs, which overlapped with antigenic regions recognized by T cells in the peripheral blood of T1D patients. T cell antigen specificity to these proinsulin regions provide an avenue for developing biomarkers in the peripheral blood that mirror the islets. The level of T cell response to proinsulin epitopes was lower than that observed with an influenza-specific TCR, but over half of the TCRs responded to native proinsulin peptides as comparably as a level of optimal response exhibited by a TCR specific in another autoimmune disease (celiac disease). Biological targets for the TCRs with low responses may be neoepitopes modified from the natural form of proinsulin.

## Materials and Methods

### T Cell Receptor Transductants

TCR sequences were identified as described previously (10). TCR transductants were generated using a recently published protocol (38). Briefly, 5KC T-hybridoma cells (56) were transduced with a NFAT-driven fluorescent reporter, ZsGreen-1, along with the human CD4 gene with two amino acid mutations at positions 40 (glutamine to tyrosine) and 45 (threonine to tryptophan) that increase binding to MHC molecules (57) (the retroviral vector is available from addgene, plasmid ID 162745) using a standard spinfection protocol with viral supernatant produced from phoenix-eco cells (ATCC CRL-3214) (58). Cells were also transduced with a combination of two fluorescent protein genes (addgene plasmid ID 153423, 153424, 153524, 153426, 153427, 153428) as an identifier of each TCR. We then transduced each 5KC cell line expressing a specific combination of fluorescent proteins with a retroviral vector encoding a chimeric TCR alpha gene followed by a porcine teschovirus-1 2A (P2A) peptide and a chimeric TCR beta gene (58).

### Screening of T Cell Receptor Transductants for Reactivity to Preproinsulin

Up to eight TCR transductants (20,000 cells per line) expressing different combinations of fluorescent proteins were pooled and cultured with a preproinsulin truncated peptide pool in the presence of autologous B cells transformed with EBV (100,000 cells per well) in a well of round-bottom 96-well-plates. The preproinsulin truncated peptide pools were purchased from Mimotopes (Mulgrave, Australia) as pools of four crude peptides containing 3-7 mg peptides per pool. Each peptide pool was dissolved in 250 µl 80% dimethylsulfoxide/20% water, and 2 µl of dissolved peptide pool was added in a culture well containing total 200 µl media; thus the peptide concentration was approximately 200 µg/ml. Peptide sequences contained in each pool are shown in **Supplementary Table 2**. Autologous EBV-transformed B cell lines were made from spleen cells of individual islet donors using a standard protocol (59). Cultured cells were harvested next day and analyzed for expression of ZsGreen-1 in each TCR transductant line using a flow cytometer (Cytoflex, Beckman Coulter) and FlowJo (BD) (38). An example of gating strategy is shown in **Supplementary Figure 3**. Cells cultured in the presence or absence of 5 µg/ml of anti-mouse CD3ϵ antibody (Clone 125-2C11, BD) were included in the assay as positive and negative controls, respectively. The threshold for positive ZsGreen-1 expression was determined such that the majority of cells in negative control wells for all TCR transductants in a same pool becomes negative (**Supplementary Figure 3**). When the proportion of ZsGreen-1-positive cells in a negative control culture well exceeded 20% or in a positive control culture was lower than 50%, the TCR clonotypes were excluded from analysis, resulting in total 187 TCR clonotypes evaluated for the response to preproinsulin truncated peptide pools.

### Determining Optimal Epitope Regions

For TCR transductants that responded to more than one truncated peptide pools in the screening, responses against lower amounts of truncated peptide pools (x10, x100, and x1,000 dilutions of peptide solutions used for the screening) were examined to identify the most preferred pools (**Supplementary Figure 1**). 15-mer peptides contained in top 4 or 5 peptide pools that most efficiently stimulated individual TCR transductants were newly synthesized by Genemed synthesis (San Antonio, USA) (**Supplementary Table 3**). TCR transductants (20,000 cells per well) were cultured with different concentrations (10 nM, 100 nM, 1 µM, 10 µM, and 100 µM) of peptides in the presence of autologous EBV-transformed B cell lines (100,000 cells per well). After overnight culture, cells were harvested and analyzed for ZsGreen-1 expression using a flow cytometer (Cytoflex, Beckman Coulter) and FlowJo (BD) (38).

### HLA Typing

HLA-DRB1, DQA1, DQB1, DPA1, and DPB1 alleles were determined using DNA samples extracted from spleen cells of each donor by the Barbara Davis Center Autoantibody/HLA Core Facility (https://medschool.cuanschutz.edu/barbara-davis-center-for-diabetes/service-centers/autoantibody-hla-service-center). To determine DRB3 and DRB4 alleles of the donor nPOD 6414, we PCR-amplified 273-294 bp fragments in which 96-185 bp were overlapped with adjacent fragments at each end using sets of primers as shown in **Supplementary Table 5**. The amplicons were connected to Illumina adaptors by 8 cycles of PCR using a set of primers (CAAGCAGAAGACGGCATACGAGAT-CGTGAT (Index)-GTGACTGGAGTTCAGACGTGTGCTCTTCCGATCT & AATGATACGGCGACCACCGAGATCT-ACACTCTTTCCCTACACGACGCTCTTCCGATCT), followed by sequencing on a NovaSEQ sequencer (Illumina). Contig sequences were blasted to the IMGT/HLA database (https://www.ebi.ac.uk/Tools/services/web_ncbiblast/toolform.ebi?tool=ncbiblast&context=nucleotide&database=imgthla) to identify alleles of DRB3 and DRB4 genes.

### HLA Transductants

Two types of antigen presenting cells were generated using the retroviral or lentiviral expression system. For the initial analysis to determine HLA-DR, DQ, or DP (experiments shown in left panels of **Figures 3**–**5**, and **6A**), retroviral vectors encoding a puromycin resistance gene along with a first set of DRA1-P2A-DRB1, DQA1-P2A-DQB1, or DPA1-P2A-DPB1 gene cassette were generated for each donor, and transduced into K562 cells (ATCC CCL-243) using a standard spinfection protocol with viral supernatant produced from 293T cells (ATCC CRL-3216) (58), followed by sorting of cells stained with anti-HLA-DR antibody conjugated with allophycocyanin (clone L243, BioLegend), anti-HLA-DQ antibody conjugated with phycoerythrin (clone REA303, Miltenyi BIotec), or anti-HLA-DP antibody conjugated with allophycocyanin (clone B7/21, Leinco Technologies). Retroviral vectors encoding an E2-Crimson fluorescent protein gene along with another set of DRA1-P2A-DRB1, DQA1-P2A-DQB1, or DPA1-P2A-DPB1 gene cassettes were generated for each donor, and transduced into K562 cells that have been transduced with the first set of DR, DQ, or DP gene cassette, followed by sorting of cells expressing E2-Crimson. For all subsequent experiments, lentiviral vectors encoding a DRA1-P2A-DRB1, DQA1-P2A-DQB1, DPA1-P2A-DPB1, DRA1-P2A-DRB3, or DRA1-P2A-DRB4 gene cassette were generated and transduced into K562 cells (ATCC CCL-243) using a standard spinfection protocol with viral supernatant produced from 293FT cells (Thermo Fisher Scientific). Expressions of HLA molecules were analyzed using the antibodies described above. Over 97% of cells in all cell lines except the combinations of DQA1*01:01 & DQB1*03:02 and DQA1*03:01 & DQB1*05:01 (DQ trans-combinations of donor nPOD 6342) were stained with the antibodies, and therefore no sorting was needed. K562 cells transduced with the two HLA allele combinations above failed to express HLA molecules on cell surface. This observation was consistent with a previous report characterizing preferred and failed HLA alpha and beta chain combinations (60), and therefore these two trans-combinations were excluded from analysis of TCRs derived from the nPOD 6342 donor.

### Determining HLA Restrictions

TCR transductants (20,000 cells per well) were cultured with an optimal 15-mer peptide (100 µM) in the presence of K562 cells expressing appropriate HLA molecules (50,000 cells per well) in a well of round-bottom 96-well-plates. Peptide sequences used for individual TCR transductant lines are included in **Table 2**. HLA allele combinations used for individual assays are designated in figure legends. After overnight culture, cells were harvested and analyzed for ZsGreen-1 expression using a flow cytometer (Cytoflex, Beckman Coulter) and FlowJo (BD) (38). Cells cultured in the presence or absence of 5 µg/ml of anti-mouse CD3ϵ antibody (Clone 125-2C11, BD) were included in an assay as positive and negative controls, respectively. For the TCR clonotype 55.C10, TCR transductants (20,000 cells per well) were cultured with the cognate peptide (100 µM) in the presence of an EBV-transformed cell line generated from nPOD 6414 spleen cells with or without anti-HLA-DR antibody (clone L243, BD), anti-HLA-DQ antibody (generated in the Michels laboratory), or HLA-DP antibody (clone B7/21, Abcam) at 1 µM, followed by analysis of ZsGreen-1 expression next day.

### Dose–Response Assessment

TCR transductants (20,000 cells per well) were cultured with a peptide designated in **Table 2** at different concentrations (10 pM, 100 pM, 1 nM, 10 nM, 100 nM, 1 µM, 10 µM, and 100 µM) in the presence of K562 cells expressing HLA molecules designated in figure legends (50,000 cells per well), followed by analysis of ZsGreen-1 expression next day. Peptide sequences tested for the response by TCRs HA1.17 and 489 are PKYVKQNTLKLAT (influenza HA:306–318), SG**Q**GSFQPSQ**Q**NP (native α-gliadin), and SG**E**GSFQPSQ**E**NP (deamidated α-gliadin p1E,p9E). EC_50_ values were calculated using Prism 8 (GraphPad), the nonlinear regression log (agonist) vs. response (three parameters) equation model. For TCR clonotypes, 6.G4 and 23.G8, EC_50_ values were not determined due to weak responses, and therefore the nonlinear regression log (agonist) vs. normalized response equation model was used to determine EC_50_ values.

### Responses to Proinsulin and Human Islet Lysates

Primary islet tissues isolated from non-diabetic organ donors were obtained from the Integrated Islet Distribution Program (IIDP). Islet lysates were generated by three steps of extractions using trifluoroacetic acid, ammonium bicarbonate, and trifluoroethanol. Extracts were lyophilized and dissolved in urea. K562 cells expressing cognate HLA molecules for each TCR clonotype (50,000 cells per well) were cultured with proinsulin (Amidebio LLC, Louisville, CO) at 200 µg/ml or lysates generated from 600-1600 IEQs of primary islets overnight. TCR transductants (20,000 cells per well) were directly added to culture wells containing K562 cells pulsed with proinsulin or islet lysates, followed by analysis of ZsGreen-1 expression next day.

### Responses to Native and Citrullinated Peptides

TCR transductants (20,000 cells per well) were cultured with native insulin B-chain peptide (SHLVEALYLVCGE-**R**-GFFYTPK) or a citrullinated insulin B-chain peptide (SHLVEALYLVCGE-**Cit**-GFFYTPK) at different concentrations (100 pM, 1 nM, 10 nM, 100 nM, 1 µM, and 10 µM) in the presence of K562 cells expressing HLA molecules designated in figure legends (50,000 cells per well), followed by analysis of ZsGreen-1 expression next day.

### Computational Simulation of Predicting Core Epitopes

The IEDB MHC-II Binding Prediction online tool (http://tools.iedb.org/mhcii/) was used to predict core epitopes. Up to five 15-mers of peptides that were contained in peptide pools stimulating individual TCR transductants were analyzed using the IEDB recommended prediction 2.22 method for binding to cognate HLA molecules.

## Data Availability Statement

The original contributions presented in the study are included in the article/**Supplementary Material**. Further inquiries can be directed to the corresponding author.

## Ethics Statement

The studies involving human participants were reviewed and approved by the University of Florida Institutional Research Board (IRB201600029). Written informed consent to participate in this study was provided by the participants’ legal guardian/next of kin.

## Author Contributions

MN and AM designed and oversaw the studies. LL and AA conducted the experiments, and LL, AA, and MN analyzed the data. HR, LY, SK, MA, and CM provided essential information and materials. MN wrote the manuscript, and all authors reviewed and edited the manuscript. All authors contributed to the article and approved the submitted version.

## Funding

This work was supported by the National Institutes of Diabetes and Digestive and Kidney Diseases [grant numbers R01DK099317 (MN and AWM), R01DK032083 (MN and AWM), R01DK108868 (AWM), DP3DK110845 (MN and AWM), UC4DK104223 (MN and AWM), P30 DK116073-01A1 (MN, AWM, and HAR), UC4 DK116284 (SCK), UG3 DK122638-01 (CEM), P01 AI042288 (MAA and CEM), UC4 DK104194-01 (CEM), R01 DK120444 (HAR), R21 AI140044 (HAR), HIRN new investigator award (HAR)], JDRF [grant numbers 2-SRA-2018-480-S-B (MN and AWM), 1-SRA-2020-911-A-N (MN), 2-SRA-2019-781-S-B (HAR)], the Leona M. and Harry B. Helmsley Charitable Trust (MAA), Children’s Diabetes Foundation (HAR), and the Culshaw Family Junior Investigator Award (MN and HAR). SCK is the George F. and Sybil H. Fuller Term Chair in Diabetes.

## Conflict of Interest

The authors declare that the research was conducted in the absence of any commercial or financial relationships that could be construed as a potential conflict of interest.
