# Supplementary material for: Proinsulin-Reactive CD4 T Cells in the Islets of Type 1 Diabetes Organ Donors
Source: Front Endocrinol (Lausanne). 2021 Mar 25;12:622647. doi: 10.3389/fendo.2021.622647 (PMC8027116; doi:10.3389/fendo.2021.622647)
Supplement: Supplementary file 1 [file DataSheet_1.pdf]

Supplementary Figure 1

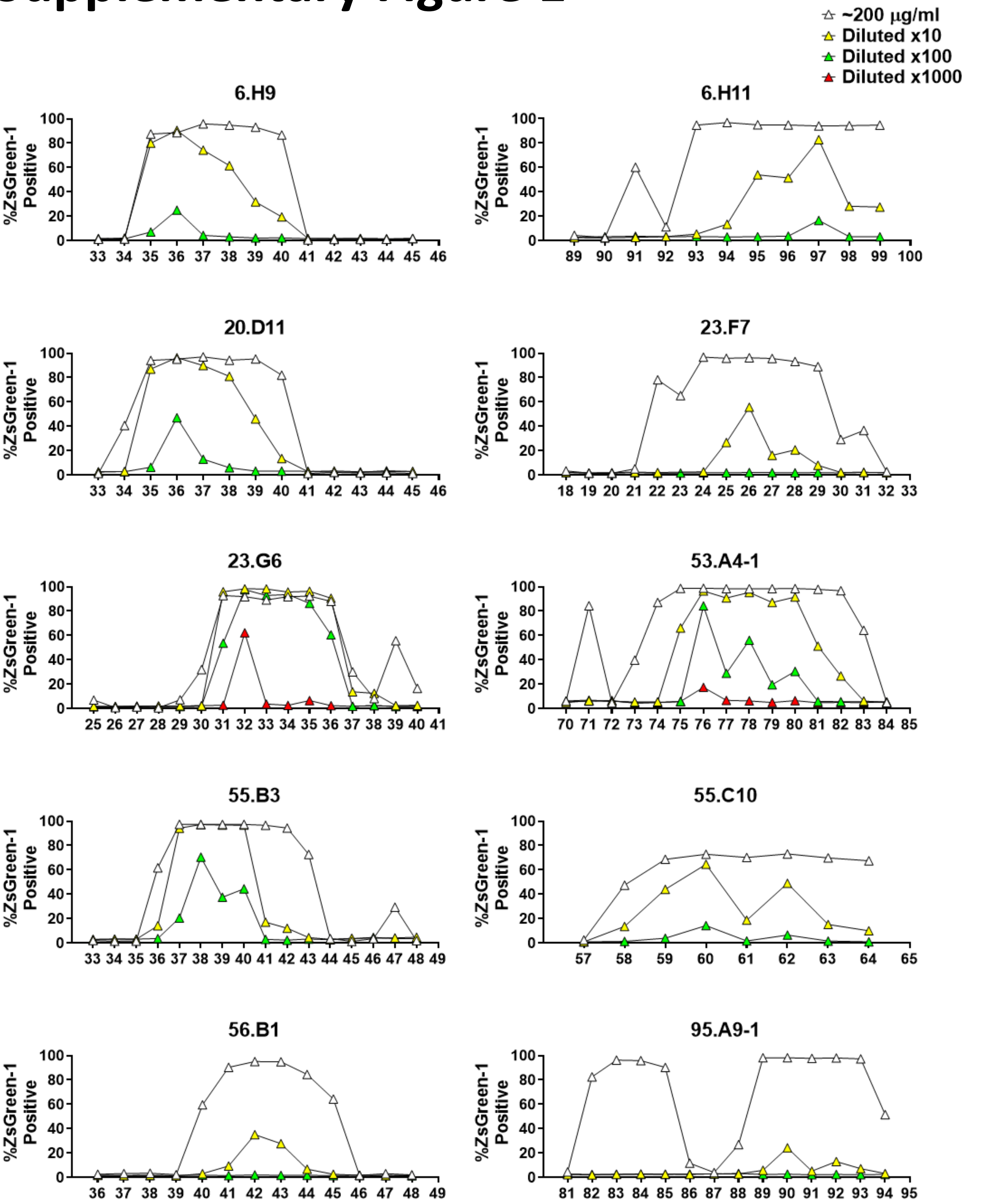

TCR transductants were cultured with peptide pools that induced positive responses by the screening examination. Peptide pools diluted at 10, 100, and 1,000 times were added to test the response by each TCR transductant in the presence of autologous EBV-transformed B cells. Percentages of ZsGreen-1-positive cells are plotted.

Supplementary Figure 2

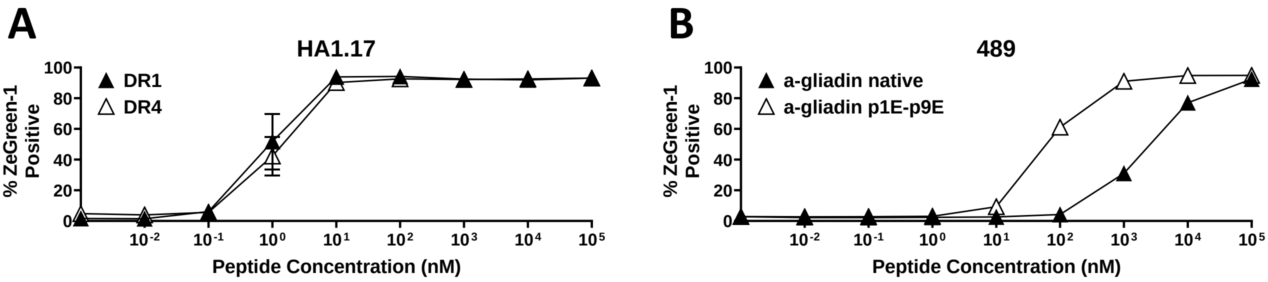

(A) The HA.1.17 TCR transductants, known to be reactive to an influenza peptide presented by DR1 or DR4, were cultured with different concentrations of cognate peptides in the presence of K562 cells transduced with DR1 [DRA1*01:01 & DRB1*01:01] (black triangles) or DR4 [DRA1*01:01 & DRB1*04:01] (white triangles). (B) The 489 TCR transductants, known to be reactive to an alpha-gliadin peptide, were cultured with different concentrations of the deamidated (white triangles) or native form (black triangles) of alpha-gliadin peptides in the presence of K562 cells transduced with DQ8 [DQA1*03:01 & DQB1*03:02]. Experiments were independently repeated three times, and mean values $\pm$ standard error of the mean are shown.

Supplementary Figure 3

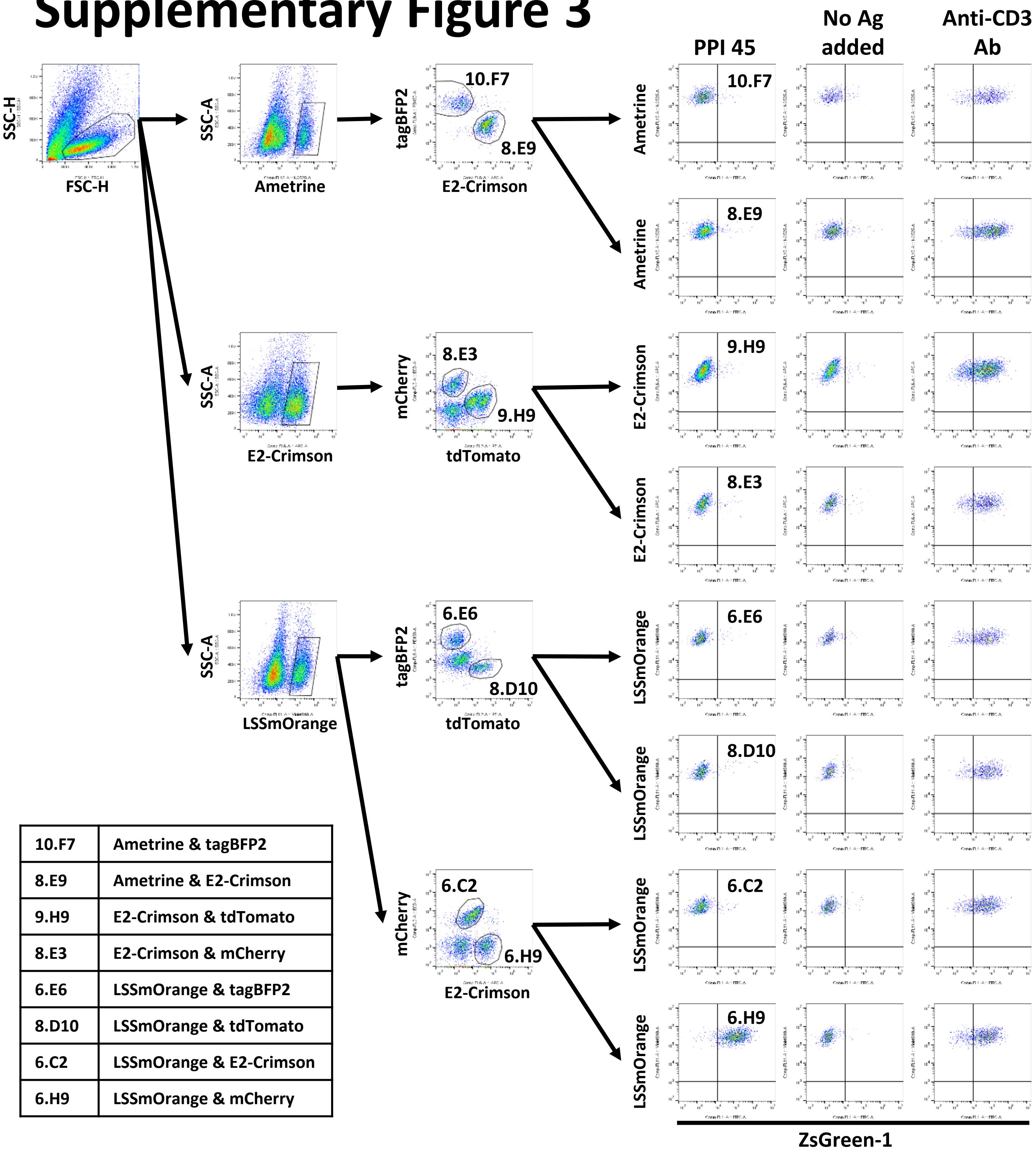

10.F7	Ametrine & tagBFP2
8.E9	Ametrine & E2-Crimson
9.H9	E2-Crimson & tdTomato
8.E3	E2-Crimson & mCherry
6.E6	LSSmOrange & tagBFP2
8.D10	LSSmOrange & tdTomato
6.C2	LSSmOrange & E2-Crimson
6.H9	LSSmOrange & mCherry

An example of gating strategy of T cell stimulation assays is shown using a sample testing a pool of 8 TCR transductants derived from an nPOD 6323 donor for the response to a peptide pool 45, which contains peptides recognized by a TCR 6.H9, in the presence of autologous EBV-transformed cells. Each TCR transductant line constitutively expresses two fluorescent proteins as identifiers. Live cells were identified in the FSC & SSC scatter plot. Each transductant line was identified in two steps by gating cells expressing a first fluorescent identifier, and then a second fluorescent identifier. Cells gated in each fluorescent combination are then evaluated for ZsGreen-1 expression. Among cells gated in LSSmOrange and mCherry (i.e. 6.H9), 96% of cells express ZsGreen-1. Flow images of same TCR transductants cultured with or without anti-mouse CD3ε antibody, which were gated using the same strategy, are included as negative and positive controls, respectively. While individual TCR transductant pools contained different combinations of fluorescent identifiers. each TCR transductant line was identified using a similar gating strategy.
