# Supplementary material for: Proinsulin-Reactive CD4 T Cells in the Islets of Type 1 Diabetes Organ Donors
Source: Front Endocrinol (Lausanne). 2021 Mar 25;12:622647. doi: 10.3389/fendo.2021.622647 (PMC8027116; doi:10.3389/fendo.2021.622647)
Supplement: Supplementary file 2 [file DataSheet_2.pdf]

Supplementary Table 1: TCR sequences analyzed

Donor ID	TCR ID	TRAV	TRAJ	CDR3a	TRBV	TRBJ	CDR3b
nPOD69	1.A1	TRAV5	TRAJ29	CAETRNSGNTPLVF	TRBV12-5	TRBJ2-6	CASGRFGANVLTF
	1.A11	TRAV39	TRAJ58	CAVDVETSGSRLTF	TRBV20-1	TRBJ2-1	CSARVLAGARNNEQFF
	1.A12	TRAV19	TRAJ57	CALSEATQGGSEKLVF	TRBV7-2	TRBJ2-5	CASSLLAGVGKTQYF
	1.A2	TRAV16	TRAJ28	CALHSGAGSYQLTF	TRBV28	TRBJ2-3	CASSIGASTDTQYF
	1.A4	TRAV19	TRAJ56	CALSEVSAGANSKLTF	TRBV18	TRBJ1-5	CASSPLGNQPQHF
	1.A7	TRAV36/DV7	TRAJ47	CAVGEYGNKLVF	TRBV27	TRBJ2-4	CASSLSRGGANIQYF
	1.A8	TRAV36/DV7	TRAJ39	CAALNNAGNMLTF	TRBV25-1	TRBJ2-1	CASSESLAGGKQFF
nPOD6323	10.A2	TRAV12-2	TRAJ49	CAVNVNQFYF	TRBV20-1	TRBJ2-3	CSARATGGAGGTDTQYF
	10.C10	TRAV2	TRAJ28	CAVEDLGSGAGSYQLTF	TRBV12-3/12-4	TRBJ2-7	CASRKEGDAYEQYF
	10.D4-2	TRAV8-6	TRAJ12	CAVSRMDSSYKLIF	TRBV7-2	TRBJ1-1	CASSLEGRAEAFF
	10.E6	TRAV8-3	TRAJ23	CAVGEVGNQGGKLIF	TRBV7-2	TRBJ2-7	CASSFGLAQGEQYF
	10.F1	TRAV26-1	TRAJ16	CIVALSDGQKLLF	TRBV6-6	TRBJ1-1	CASSRTPNTEAFF
	10.F7	TRAV13-1	TRAJ47	CAASYGNKLVF	TRBV20-1	TRBJ2-3	CSATSGTMDTQYF
	10.G5	TRAV8-3	TRAJ8	CAVGKTGFQKLVF	TRBV4-2	TRBJ2-1	CASSQDWTGTSLGNEQFF
	11.H8	TRAV13-1	TRAJ10	CAASTGGGNKLTF	TRBV29-1	TRBJ2-5	CSVERGSLQETQYF
	6.A1	TRAV16	TRAJ13	CALKAGGYQKVTF	TRBV10-2	TRBJ1-1	CASSQGKTEAFF
	6.A4	TRAV6	TRAJ4	CALEGMGGYNKLIF	TRBV7-2	TRBJ1-1	CASSAGSEAFF
	6.B3	TRAV29/DV5	TRAJ58	CAASAGGTSGSRLTF	TRBV7-2	TRBJ1-1	CASSRRTGFRTEAFF
	6.B4	TRAV13-1	TRAJ21	CAATNFNKFYF	TRBV7-3	TRBJ2-7	CASSLEGASYEQYF
	6.C2	TRAV16	TRAJ22	CALISSGSARQLTF	TRBV5-6	TRBJ2-7	CASSSVGSYEQYF
	6.D7	TRAV9-2	TRAJ13	CALSDVGYQKVTF	TRBV12-3/12-4	TRBJ2-3	CASGDRGADTQYF
	6.E6	TRAV16	TRAJ22	CALSASSGSARQLTF	TRBV28	TRBJ2-7	CASSRSSYEQYF
	6.F12	TRAV38-2/DV8	TRAJ49	CAYRSANTGNQFYF	TRBV7-2	TRBJ1-5	CASSLFSGWNQPQHF
	6.F6	TRAV8-6	TRAJ24	CAVSAPDSWGKLQF	TRBV5-4	TRBJ2-2	CASSPDRGRTGELFF
	6.G10	TRAV13-1	TRAJ18	CAASTRGSTLGRLYF	TRBV6-2/6-3	TRBJ2-3	CASSSSDRGGTDTQYF
	6.G4	TRAV26-1	TRAJ8	CIVRVRNTGFQKLVF	TRBV27	TRBJ1-1	CASSPGPGNTEAFF
	6.G6	TRAV12-2	TRAJ32	CAVNRYGGATNKLIF	TRBV4-1	TRBJ1-6	CASSPPAQGNSPLHF
	6.H11	TRAV26-1	TRAJ13	CIVRVYSGGYQKVTF	TRBV30	TRBJ2-3	CAWSARLAGGPRTQYF
	6.H5	TRAV26-1	TRAJ20	CIVRVNDYKLSF	TRBV20-1	TRBJ2-5	CSAKGTSVQETQYF
	6.H9	TRAV26-1	TRAJ40	CIVRVDSGTYKYIF	TRBV7-2	TRBJ2-1	CASSLTAGLASTYNEQFF
	7.A10	TRAV12-1	TRAJ5	CVVNIEADWDTGRRALTF	TRBV7-9	TRBJ1-5	CASSVGSSNQPQHF
	7.A11	TRAV12-1	TRAJ53	CVVNKYPLFDSGGSNYKLTF	TRBV5-5	TRBJ2-3	CASSLSAIRDRGTDTQYF
	7.B10	TRAV3	TRAJ22	CAVRAVLSGSARQLTF	TRBV7-9	TRBJ1-2	CASSPRGQEGYTF
	7.B4	TRAV4	TRAJ42	CHHGGSQGNLIF	TRBV5-4	TRBJ1-2	CASSLLQTNYGYTF
	7.D3	TRAV17	TRAJ48	CATAIISNFGNEKLTF	TRBV18	TRBJ1-3	CASSRTTGADPGNTIYF
	7.D3-2	TRAV29/DV5	TRAJ22	CAASPLSGSARQLTF	TRBV18	TRBJ1-3	CASSRTTGADPGNTIYF
	7.E10	TRAV13-1	TRAJ49	CAASTRRGTGNQFYF	TRBV7-9	TRBJ2-6	CASSLWASGRDSSFSGANVLTF
	7.G11-1	TRDV1	TRAJ17	CALGELPIKAAGNKLTF	TRBV5-4	TRBJ1-2	CASSWLEEDSLSGGYGYTF
	7.G11-2	TRAV14/DV4	TRAJ27	CAMREAYNTNAGKSTF	TRBV5-4	TRBJ1-2	CASSWLEEDSLSGGYGYTF
	7.G4	TRAV26-1	TRAJ8	CIVRVINTGFQKLVF	TRBV6-6	TRBJ2-5	CASSRTTSGRPETQYF
	7.H5	TRAV12-1	TRAJ28	CVVIPGAGSYQLTF	TRBV27	TRBJ2-1	CASSLLGRSNEQFF
	8.B2	TRAV16	TRAJ22	CALGKGSARQLTF	TRBV9	TRBJ2-3	CASSPRLAGEITDTQYF
	8.C2	TRAV26-1	TRAJ22	CIVRSGSARQLTF	TRBV2	TRBJ2-2	CASRAESRNGELFF
	8.C3	TRAV26-1	TRAJ7	CIVRTVAGNNRLAF	TRBV3-1	TRBJ2-5	CASSQSGTQETQYF
	8.C6	TRAV38-2/DV8	TRAJ23	CAYRSYNQGGKLIF	TRBV7-9	TRBJ2-6	CASSFDSSGANVLTF
	8.D10	TRAV13-1	TRAJ23	CAANNQGGKLIF	TRBV20-1	TRBJ2-7	CSAREGTSDDEQYF
	8.D12	TRAV38-2/DV8	TRAJ23	CAYRSYNQGGKLIF	TRBV7-9	TRBJ2-6	CASSLANSGANVLTF
	8.D2	TRAV26-1	TRAJ8	CIVRAVTYNTGFQKLVF	TRBV19	TRBJ2-7	CASSMLGNTYEQYF
	8.D6	TRAV8-3	TRAJ22	CAVSSGSARQLTF	TRBV7-2	TRBJ1-5	CASSPTGNSNQPQHF
	8.E11	TRAV13-1	TRAJ26	CAASKTYGQNFVF	TRBV27	TRBJ2-3	CASSLRQGGTDTQYF
	8.E12	TRAV13-1	TRAJ45	CAAKGGGADGLTF	TRBV19	TRBJ2-7	CASSSGLAQQYF
	8.E3	TRAV2	TRAJ37	CAVDGSGNTGKLIF	TRBV4-1	TRBJ2-7	CASSQDLAGVREQYF
	8.E4	TRAV9-2	TRAJ39	CALNAGNMLTF	TRBV7-2	TRBJ2-5	CASSLNRGKETQYF
	8.E9	TRAV13-1	TRAJ20	CAASQYDYKLSF	TRBV28	TRBJ2-3	CASSSSTDTQYF
	8.F7	TRAV38-2/DV8	TRAJ15	CAYRNRGTLAGTALIF	TRBV7-9	TRBJ2-1	CASSPRTRHYEQFF

	8.G12	TRAV12-3	TRAJ4	CAQRSGGYNKLIF	TRBV7-9	TRBJ1-5	CASSVGSSNQPQHF
	8.G6	TRAV13-1	TRAJ13	CAASIGGYQKVTF	TRBV6-1	TRBJ2-1	CASSVASNEQFF
	8.H10	TRAV8-6	TRAJ54	CAVRGIIQGAQKLVF	TRBV5-4	TRBJ2-6	CASTLLGSGANVLTF
	8.H11	TRAV12-2	TRAJ13	CAVGMGYQKVTF	TRBV10-2	TRBJ1-2	CASRDLQGGRNYGYTF
	8.H6	TRAV27	TRAJ33	CAGLDSNYQLIW	TRBV7-2	TRBJ2-1	CASSLGVAYNEQFF
	8.H8	TRAV26-1	TRAJ32	CTDRGATNKLIF	TRBV18	TRBJ1-6	CASSSERRNSPLHF
	9.D3	TRAV8-6	TRAJ27	CAVIPFGGNAGKSTF	TRBV4-1	TRBJ1-4	CASSQVGQGTNEKLFF
	9.H9	TRAV9-2	TRAJ13	CALPSGGYQKVTF	TRBV28	TRBJ2-7	CASSQHQRTGGEQYF
nPOD6342	18.B7	TRAV17	TRAJ13	CATALYSGGYQKVTF	TRBV5-1	TRBJ2-3	CASSWTGGATDTQYF
	18.D6	TRAV3	TRAJ45	CAATGGGADGLTF	TRBV19	TRBJ2-2	CASSTTRGQGVRRNTGELFF
	18.D7	TRAV13-2	TRAJ37	CAASGGSSNTGKLIF	TRBV18	TRBJ2-1	CASSPGADDNEQFF
	18.F12	TRAV21	TRAJ42	CAVNYGGSQGNLIF	TRBV19	TRBJ1-5	CASSIDRNQPQHF
	18.H7	TRAV12-1	TRAJ13	CVVNTYSGGYQKVTF	TRBV19	TRBJ2-7	CASSPRPLTYEQYF
	19.A4	TRAV8-6	TRAJ32	CAVRETGATNKLIF	TRBV20-1	TRBJ2-7	CSARPQGFSSYEQYF
	19.D1	TRAV13-1	TRAJ26	CAASLTHGQNFVF	TRBV11-2	TRBJ2-7	CASSPPGLGEQYF
	19.D12	TRAV38-2/DV8	TRAJ58	CAYRSGETSGSRLTF	TRBV4-2	TRBJ2-5	CASSLVASGAVQETQYF
	19.E11	TRAV12-1	TRAJ28	CVVIPAGAGSYQLTF	TRBV5-4	TRBJ2-1	CASSPRDGSSYNEQFF
	19.E9	TRAV20	TRAJ22	CAVAGSARQLTF	TRBV5-4	TRBJ2-1	CASSPRDGSSYNEQFF
	19.H4	TRAV9-2	TRAJ28	CALSDRSGAGSYQLTF	TRBV18	TRBJ2-6	CASSPPAGDRLTSGANVLTF
	20.A1	TRAV13-2	TRAJ40	CAETQSTSGTYKYIF	TRBV28	TRBJ2-1	CASSLPGGAGNEQFF
	20.C1	TRAV27	TRAJ27	CAGAESGANAGKSTF	TRBV3-1	TRBJ1-1	CASKRASMNTEAFF
	20.D11	TRAV12-3	TRAJ4	CAILSGGYNKLIF	TRBV2	TRBJ2-5	CASSAETQYF
	20.E4	TRAV13-1	TRAJ12	CAAKSSYKLIF	TRBV19	TRBJ1-1	CASSAPLNRDAEAFF
	20.F4	TRAV6	TRAJ39	CALDIPEAGNMLTF	TRBV5-4	TRBJ2-1	CASSLLAGDNEQFF
	21.B2	TRAV14/DV4	TRAJ34	CAMRGNTDKLIF	TRBV11-1	TRBJ1-3	CASSLHRARSGNTIYF
	21.B8	TRAV21	TRAJ49	CAVRHNGNQFYF	TRBV29-1	TRBJ2-3	CSVNRGGTDTQYF
	21.E1	TRAV1-1	TRAJ29	CAVRDSGNTPLVF	TRBV30	TRBJ1-3	CAWRLTGGSGNTIYF
	21.E3	TRAV3	TRAJ23	CAVRDKGNQGGKLIF	TRBV6-5	TRBJ2-7	CASSLNRMAYEQYF
	21.G1	TRAV13-1	TRAJ17	CAASIKAAGNKLTF	TRBV20-1	TRBJ1-3	CSARGPNSGNTIYF
	21.H11	TRAV13-1	TRAJ22	CAATFPPGSARQLTF	TRBV2	TRBJ1-4	CASRDRGGGEKLFF
	21.H11-2	TRAV8-3	TRAJ33	CAVGESNYQLIW	TRBV2	TRBJ1-4	CASRDRGGGEKLFF
	22.C12	TRAV12-1	TRAJ29	CVVEVSGNTPLVF	TRBV12-3	TRBJ1-1	CASSFESRAFF
	22.C12-2	TRAV14/DV4	TRAJ18	CAMRDPFDRGSTLGRLYF	TRBV12-3	TRBJ1-1	CASSFESRAFF
	22.C6	TRAV22	TRAJ28	CAVGRYSGAGSYQLTF	TRBV6-1	TRBJ1-2	CASSDGTGPYGYTF
	22.D3	TRAV13-1	TRAJ8	CAASRAGFQKLVF	TRBV27	TRBJ1-6	CASSPPPGGNSPLHF
	23.B4	TRAV8-3	TRAJ31	CAVGAWNNNARLMF	TRBV6-5	TRBJ1-6	CASSYGTVNSPLHF
	23.C10	TRAV1-1	TRAJ6	CAPRVGSYIPTF	TRBV30	TRBJ1-3	CAWRLTGGSGNTIYF
	23.C11	TRAV12-1	TRAJ13	CVVNRYSGGYQKVTF	TRBV9	TRBJ2-5	CASSMTSGVSETQYF
	23.D8-2	TRAV8-1	TRAJ40	CAVSTTSGTYKYIF	TRBV20-1	TRBJ1-2	CSARGPTAHGYTF
	23.E10	TRAV4	TRAJ4	CLVGAPISGGYNKLIF	TRBV24-1	TRBJ2-5	CATSDLQMGASRETQYF
	23.E10-2	TRAV12-2	TRAJ9	CAVTGGFKTIF	TRBV24-1	TRBJ2-5	CATSDLQMGASRETQYF
	23.F7	TRAV8-2	TRAJ29	CAVIASGNTPLVF	TRBV19	TRBJ2-3	CASKGPGTVIRADTQYF
	23.G6	TRAV9-2	TRAJ6	CALSISGGSYIPTF	TRBV5-1	TRBJ2-5	CASSFRQGEQETQYF
	23.G8	TRAV39	TRAJ56	CAWRTGANSKLTF	TRBV24-1	TRBJ2-2	CATGLAANTGELFF
	24.A7	TRAV21	TRAJ49	CAVRFPGNQFYF	TRBV29-1	TRBJ2-3	CSVNRGGTDTQYF
	24.D8	TRAV8-4	TRAJ27	CAVTLNTNAGKSTF	TRBV7-3	TRBJ2-2	CASSPIINTGELFF
	24.E5	TRAV8-6	TRAJ15	CAMKTQGQAGTALIF	TRBV19	TRBJ2-2	CASSISNTGELFF
	25.D11	TRAV9-2	TRAJ17	CALSASWTAGNKLTF	TRBV20-1	TRBJ2-1	CSARATASGAYNEQFF
nPOD6367	26.A2	TRAV8-2/8-4	TRAJ24	CVVRATDSWGKFQF	TRBV11-2	TRBJ2-3	CASSLGAGPADTQYF
	26.A5	TRAV24	TRAJ50	CAPQSGGNKVIF	TRBV6-5	TRBJ1-1	CASSYRGERAAFF
	26.B3-1	TRAV23/DV6	TRAJ32	CAASPNYGGATNKLIF	TRBV28	TRBJ1-6	CASSLARGSPLHF
	26.B3-2	TRAV21	TRAJ47	CAVRREAEYGNKLVF	TRBV28	TRBJ1-6	CASSLARGSPLHF
	26.D8	TRAV8-2/8-4	TRAJ30	CAVGYLNRDDKIIF	TRBV12-3/12-4	TRBJ1-5	CASSSDDQPQHF
	28.H2	TRAV27	TRAJ49	CAGEAFYF	TRBV9	TRBJ1-3	CASSPMWGNTIYF
	29.D4-1	TRAV38-2/DV8	TRAJ48	CAHAPNFGNEKLTF	TRBV6-1	TRBJ1-5	CASSGRASLLQPQHF
	29.F8	TRAV13-1	TRAJ23	CAASEGGKLIF	TRBV6-2/6-3	TRBJ1-1	CASSFVGDTEAFF
	29.G4	TRAV24	TRAJ49	CAFIRSGNQFYF	TRBV12-3/12-4	TRBJ2-3	CASSFSDNTDTQYF
	30.H8-1	TRAV23/DV6	TRAJ12	CAASLINKMDSSYKLIF	TRBV2	TRBJ1-1	CASSEGRQRAFF

	30.H8-2	TRAV26-1	TRAJ48	CIVRSNFGNEKLTF	TRBV2	TRBJ1-1	CASSEGRQRAFF
nPOD6414	53.A4-1	TRAV39	TRAJ33	CAVDPMDSNYQLIW	TRBV29-1	TRBJ2-6	CSVGTDPSGANVLTF
	53.A4-2	TRAV13-1	TRAJ43	CAASKNNNNDMRF	TRBV29-1	TRBJ2-6	CSVGTDPSGANVLTF
	55.B10	TRAV34	TRAJ45	CGADMARSGGGADGLTF	TRBV29-1	TRBJ2-1	CSAGHNEQFF
	55.B3	TRAV21	TRAJ29	CAVLPPTPLVF	TRBV18	TRBJ1-1	CASSYPGTGGARTEAFF
	55.B6	TRAV13-1	TRAJ15	CAASGGGTALIF	TRBV5-1	TRBJ2-2	CASSLELAGRKHTGELFF
	55.C10	TRAV26-1	TRAJ26	CIVRSHGQNFVF	TRBV20-1	TRBJ2-7	CSARPGTRNYEQYF
	55.C3	TRAV9-2	TRAJ42	CALRGGGSQGNLIF	TRBV2	TRBJ2-3	CASSEWTSTDTQYF
	55.D6	TRAV26-1	TRAJ37	CIVRPLLSSNTGKLIF	TRBV20-1	TRBJ1-6	CSARDGEGGDSPLHF
	55.E10	TRAV12-2	TRAJ45	CAVTRGGADGLTF	TRBV7-8	TRBJ2-5	CASSLGWGETQYF
	55.E4	TRAV8-2/8-4	TRAJ21	CAGNNFNKFYF	TRBV7-9	TRBJ1-1	CASSSRREDTEAFF
	55.E5	TRAV19	TRAJ3	CALSEGGSSASKIIF	TRBV29-1	TRBJ1-1	CSVEDSWSGEAFF
	55.E5-2	TRAV12-1	TRAJ5	CVVNMVNTGRRALTF	TRBV29-1	TRBJ1-1	CSVEDSWSGEAFF
	55.F11	TRAV13-1	TRAJ10	CAAATGGGNKLTF	TRBV6-6	TRBJ1-5	CASRRTGGQSNQPQHF
	55.F2	TRAV39	TRAJ24	CAGIDSWGKLQF	TRBV6-2	TRBJ2-7	CASSYRRGTYEQYF
	55.G4	TRAV41	TRAJ53	CAVRQESGGSNYKLTF	TRBV3-1	TRBJ2-3	CASSHKTGTTDTQYF
	55.G9	TRAV34	TRAJ40	CGAHKAGTYKYIF	TRBV4-3	TRBJ2-5	CASSPFSAGQEETQYF
	55.H6-1	TRAV26-1	TRAJ48	CIVSPNFGNEKLTF	TRBV20-1	TRBJ2-1	CSARDSIGLGSEQFF
	55.H6-2	TRAV26-1	TRAJ47	CIVRVAGKYGNKLVF	TRBV20-1	TRBJ2-1	CSARDSIGLGSEQFF
	56.A9	TRAV26-1	TRAJ12	CIVSPDSSYKLIF	TRBV5-1	TRBJ1-5	CASSPGRSNQPQHF
	56.B1	TRAV13-1	TRAJ40	CAVLSPSGTYKYIF	TRBV7-9	TRBJ1-4	CASSLMGNPHEKLFF
	56.B3-1	TRAV20	TRAJ52	CAVDGYGKLTF	TRBV6-1	TRBJ1-5	CASSSVGGGGQPQHF
	56.B8	TRAV13-1	TRAJ48	CAAIPFGNEKLTF	TRBV27	TRBJ2-3	CASSPTRLLAGGGDTQYF
	56.C4	TRAV26-1	TRAJ37	CIVTSGNTGKLIF	TRBV29-1	TRBJ2-5	CSVDSSAREETQYF
	56.C8	TRAV8-3	TRAJ45	CAVVRMGGGADGLTF	TRBV5-1	TRBJ2-5	CASSVAGGEETQYF
	56.G8	TRAV26-1	TRAJ45	CIVIGSGGGADGLTF	TRBV6-5	TRBJ2-7	CASSKTGTGSYEQYF
	56.H10	TRAV20	TRAJ45	CAGTGGGADGLTF	TRBV7-2	TRBJ1-5	CASSPRRGGGGQPQHF
	56.H3-1	TRAV12-1	TRAJ24	CVVNKPDSWGKLQF	TRBV7-2	TRBJ2-5	CASSRPGYLQETQYF
	56.H3-2	TRAV17	TRAJ34	CATGPMNADKLIF	TRBV7-2	TRBJ2-5	CASSRPGYLQETQYF
	56.H4-1	TRAV4	TRAJ31	CLVGDKRNNARLMF	TRBV6-1	TRBJ2-3	CASSEARGEYF
	56.H4-2	TRAV12-2	TRAJ7	CAVNIYGNNRLAF	TRBV6-1	TRBJ2-3	CASSEARGEYF
	57.A11	TRAV26-1	TRAJ40	CIVRSHSGTYKYIF	TRBV5-1	TRBJ1-1	CASSLMTGNTEAFF
	57.A3	TRAV26-1	TRAJ23	CIVRVERQGGKLIF	TRBV4-2	TRBJ2-7	CASSKAGTGYYEQYF
	57.B4	TRAV14/DV4	TRAJ53	CAMREGPRLGGSNYKLTF	TRBV19	TRBJ2-3	CASSIGGGPHTDTQYF
	57.C7	TRAV26-1	TRAJ17	CIVKEAAGNKLTF	TRBV20-1	TRBJ1-1	CSAPRGGELNTEAFF
	57.D5	TRAV13-1	TRAJ17	CAASIKAAGNKLTF	TRBV20-1	TRBJ1-3	CSARGPNSGNTIYF
	57.E4	TRAV2	TRAJ6	CAVEEGGSYIPTF	TRBV2	TRBJ2-1	CASAEGPAGTSAPDPF
	57.F12-1	TRAV2	TRAJ26	CAMTHFRDNYGQNFVF	TRBV4-3	TRBJ2-1	CASSQDRGLAGNNEQFF
	57.F12-2	TRAV21	TRAJ31	CAGLNNNARLMF	TRBV4-3	TRBJ2-1	CASSQDRGLAGNNEQFF
	57.F3	TRAV2	TRAJ6	CAVEEGGSYIPTF	TRBV2	TRBJ2-7	CASSEAPAGASYEQYF
	57.H8	TRAV12-3	TRAJ35	CAMSARGFGNVLHC	TRBV29-1	TRBJ2-5	CSVEGRETQYF
nPOD6472	92.F1	TRAV36/DV7	TRAJ23	CAVDAVNQGGKLIF	TRBV2	TRBJ1-4	CASSELGATNEKLFF
	92.G9	TRAV12-2	TRAJ23	CAVKNNQGGKLIF	TRBV4-1	TRBJ1-1	CASSLSIVGISEAFF
	93.B6	TRAV13-1	TRAJ22	CAAGGSARQLTF	TRBV20-1	TRBJ2-3	CSVRGEADTQYF
	93.D8	TRAV23/DV6	TRAJ36	CAAPKTGANNLFF	TRBV11-2	TRBJ1-1	CASSFQGPVTEAFF
	93.F9	TRAV13-1	TRAJ33	CAASKGNNYQLIW	TRBV5-1	TRBJ2-2	CASSLSGQGDTGELFF
	94.C3	TRAV12-2	TRAJ3	CAVRHSSASKIIF	TRBV6-2/6-3	TRBJ2-1	CATQGGRGSYNEQFF
	94.C6	TRAV38-2/DV8	TRAJ32	CAYRSAHGGATNKLIF	TRBV2	TRBJ2-4	CASTYDILADIQYF
	94.D1	TRAV4	TRAJ37	CLVDRSSNTGKLIF	TRBV4-2	TRBJ1-6	CASSQGGVGNSPLHF
	94.E9	TRAV8-2	TRAJ30	CVVSQGNRDDKIIF	TRBV2	TRBJ2-7	CASRVSTYEQYF
	94.F1	TRAV9-2	TRAJ22	CALSTGSARQLTF	TRBV29-1	TRBJ2-1	CSLRAGGNNNEQFF
	94.F8	TRAV38-1	TRAJ43	CAFMKYNNNDMRF	TRBV30	TRBJ2-3	CAWSVRQGTTDTQYF
	94.G1	TRAV26-1	TRAJ30	CIVRPMNRDDKIIF	TRBV20-1	TRBJ2-1	CSATSGGFYNEQFF
	94.H9	TRAV17	TRAJ17	CATAKAAGNKLTF	TRBV3-1	TRBJ2-5	CASSHTGTGQETQYF
	95.A6	TRAV8-6	TRAJ20	CAVKSSNDYKLSF	TRBV5-1	TRBJ2-5	CASSPGTAGDTQYF
	95.A9-1	TRAV9-2	TRAJ18	CALRTDRGSTLGRLYF	TRBV11-2	TRBJ1-6	CASSLQSSYNSPLHF
	95.A9-2	TRAV9-2	TRAJ18	CALRTDRGSTLGRLYF	TRBV7-8	TRBJ1-3	CASSQHQGPGNTIYF
	95.B12	TRAV29/DV5	TRAJ52	CAAPRGGGTSYGKLTF	TRBV5-6	TRBJ2-7	CASGAGSTYEQYF

	95.E9	TRAV12-3	TRAJ52	CAMSARPGGTSYGKLTF	TRBV7-8	TRBJ1-1	CASSLQGGSNTEAFF
	95.F6	TRAV9-2	TRAJ35	CALIGFGNVLHC	TRBV27	TRBJ1-2	CASSLSYSYRGNYGYTF
	95.G6	TRAV8-1	TRAJ35	CAVNAGGFGNVLHC	TRBV19	TRBJ2-3	CASSTGLATFTDTQYF
	96.B2	TRAV8-3	TRAJ38	CAAGPLNAGNNRKLIW	TRBV2	TRBJ1-1	CASSDNAEAFF
	96.B7	TRAV13-1	TRAJ32	CAASRGGATNKLIF	TRBV6-6	TRBJ1-4	CASSYSGDSNEKLFF
	96.G10	TRAV9-2	TRAJ37	CALRIGNTGKLIF	TRBV20-1	TRBJ2-1	CSARDRGGASYNEQFF
	97.F7	TRAV26-1	TRAJ45	CIVSLDSGGGADGLTF	TRBV5-8	TRBJ2-2	CASSWANTGELFF
	98.B4-1	TRAV12-2	TRAJ6	CAVRVSGGSYIPTF	TRBV6-2/6-3	TRBJ2-6	CASSYPIGTGLSGANVLTF
	98.B4-2	TRAV20	TRAJ15	CAVQAQAGTALIF	TRBV6-2/6-3	TRBJ2-6	CASSYPIGTGLSGANVLTF
	98.C8	TRAV17	TRAJ30	CATERDDKIIF	TRBV2	TRBJ1-1	CASSGRDRGNTEAFF
	98.F2	TRAV29/DV5	TRAJ13	CAASGFGGYQKVTF	TRBV6-2/6-3	TRBJ1-1	CASRSHSGQGSTEAFF
	99.A7	TRAV26-1	TRAJ20	CIVRVAVNDYKLSF	TRBV7-9	TRBJ1-2	CASSSRGRRGYTF
	99.E10-1	TRDV1	TRAJ27	CALGSQKGTNAGKSTF	TRBV6-6	TRBJ1-1	CASSYSDRVNTEAFF
	99.E10-2	TRAV26-1	TRAJ47	CIVRVAYGNKLVF	TRBV6-6	TRBJ1-1	CASSYSDRVNTEAFF
	99.F2	TRAV8-1	TRAJ26	CAVNGDNYGQNFVF	TRBV28	TRBJ2-3	CASRLAGTDTQYF
	99.G3	TRAV12-1	TRAJ15	CVVSFNQAGTALIF	TRBV18	TRBJ2-7	CASSPETGIWEQYF

TCR sequence information from donors nPOD 69, nPOD 6323, and nPOD 6342 has been published in Diabetes 2017[10].

Supplementary Table 2: Preproinsulin Truncated peptide pools

Peptide Pool ID	15-mers	14-mers	13-mers	12-mers
1				MALWMRLLPLLA
2			MALWMRLLPLLAL	ALWMRLLPLLAL
3		MALWMRLLPLLALL	ALWMRLLPLLALL	LWMRLLPLLALL
4	MALWMRLLPLLALLA	ALWMRLLPLLALLA	LWMRLLPLLALLA	WMRLLPLLALLA
5	ALWMRLLPLLALLAL	LWMRLLPLLALLAL	WMRLLPLLALLAL	MRLLPLLALLAL
6	LWMRLLPLLALLALW	WMRLLPLLALLALW	MRLLPLLALLALW	RLLPLLALLALW
7	WMRLLPLLALLALWG	MRLLPLLALLALWG	RLLPLLALLALWG	LLPLLALLALWG
8	MRLLPLLALLALWGP	RLLPLLALLALWGP	LLPLLALLALWGP	LPLLALLALWGP
9	RLLPLLALLALWGPD	LLPLLALLALWGPD	LPLLALLALWGPD	PLLALLALWGPD
10	LLPLLALLALWGPDP	LPLLALLALWGPDP	PLLALLALWGPDP	LLALLALWGPDP
11	LPLLALLALWGPDPA	PLLALLALWGPDPA	LLALLALWGPDPA	LALLALWGPDPA
12	PLLALLALWGPDPAA	LLALLALWGPDPAA	LALLALWGPDPAA	ALLALWGPDPAA
13	LLALLALWGPDPAAA	LALLALWGPDPAAA	ALLALWGPDPAAA	LLALWGPDPAAA
14	LALLALWGPDPAAAF	ALLALWGPDPAAAF	LLALWGPDPAAAF	LALWGPDPAAAF
15	ALLALWGPDPAAAFV	LLALWGPDPAAAFV	LALWGPDPAAAFV	ALWGPDPAAAFV
16	LLALWGPDPAAAFVN	LALWGPDPAAAFVN	ALWGPDPAAAFVN	LWGPDPAAAFVN
17	LALWGPDPAAAFVNQ	ALWGPDPAAAFVNQ	LWGPDPAAAFVNQ	WGPDPAAAFVNQ
18	ALWGPDPAAAFVNQH	LWGPDPAAAFVNQH	WGPDPAAAFVNQH	GPDPAAAFVNQH
19	LWGPDPAAAFVNQHL	WGPDPAAAFVNQHL	GPDPAAAFVNQHL	PDPAAAFVNQHL
20	WGPDPAAAFVNQHLC	GPDPAAAFVNQHLC	PDPAAAFVNQHLC	DPAAAFVNQHLC
21	GPDPAAAFVNQHLCG	PDPAAAFVNQHLCG	DPAAAFVNQHLCG	PAAAFVNQHLCG
22	PDPAAAFVNQHLCGS	DPAAAFVNQHLCGS	PAAAFVNQHLCGS	AAAFVNQHLCGS
23	DPAAAFVNQHLCGSH	PAAAFVNQHLCGSH	AAAFVNQHLCGSH	AAFVNQHLCGSH
24	PAAAFVNQHLCGSHL	AAAFVNQHLCGSHL	AAFVNQHLCGSHL	AFVNQHLCGSHL
25	AAAFVNQHLCGSHLV	AAFVNQHLCGSHLV	AFVNQHLCGSHLV	FVNQHLCGSHLV
26	AAFVNQHLCGSHLVE	AFVNQHLCGSHLVE	FVNQHLCGSHLVE	VNQHLCGSHLVE
27	AFVNQHLCGSHLVEA	FVNQHLCGSHLVEA	VNQHLCGSHLVEA	NQHLCGSHLVEA
28	FVNQHLCGSHLVEAL	VNQHLCGSHLVEAL	NQHLCGSHLVEAL	QHLCGSHLVEAL
29	VNQHLCGSHLVEALY	NQHLCGSHLVEALY	QHLCGSHLVEALY	HLCGSHLVEALY
30	NQHLCGSHLVEALYL	QHLCGSHLVEALYL	HLCGSHLVEALYL	LCGSHLVEALYL
31	QHLCGSHLVEALYLV	HLCGSHLVEALYLV	LCGSHLVEALYLV	CGSHLVEALYLV
32	HLCGSHLVEALYLVC	LCGSHLVEALYLVC	CGSHLVEALYLVC	GSHLVEALYLVC
33	LCGSHLVEALYLVCG	CGSHLVEALYLVCG	GSHLVEALYLVCG	SHLVEALYLVCG
34	CGSHLVEALYLVCGE	GSHLVEALYLVCGE	SHLVEALYLVCGE	HLVEALYLVCGE
35	GSHLVEALYLVCGER	SHLVEALYLVCGER	HLVEALYLVCGER	LVEALYLVCGER
36	SHLVEALYLVCGERG	HLVEALYLVCGERG	LVEALYLVCGERG	VEALYLVCGERG
37	HLVEALYLVCGERGF	LVEALYLVCGERGF	VEALYLVCGERGF	EALYLVCGERGF
38	LVEALYLVCGERGFF	VEALYLVCGERGFF	EALYLVCGERGFF	ALYLVCGERGFF
39	VEALYLVCGERGFFY	EALYLVCGERGFFY	ALYLVCGERGFFY	LYLVCGERGFFY
40	EALYLVCGERGFFYT	ALYLVCGERGFFYT	LYLVCGERGFFYT	YLVCGERGFFYT
41	ALYLVCGERGFFYTP	LYLVCGERGFFYTP	YLVCGERGFFYTP	LVCGERGFFYTP

42	LYLVCGERGFFYTPK	YLVCGERGFFYTPK	LVCGERGFFYTPK	VCGERGFFYTPK
43	YLVCGERGFFYTPKT	LVCGERGFFYTPKT	VCGERGFFYTPKT	CGERGFFYTPKT
44	LVCGERGFFYTPKTR	VCGERGFFYTPKTR	CGERGFFYTPKTR	GERGFFYTPKTR
45	VCGERGFFYTPKTRR	CGERGFFYTPKTRR	GERGFFYTPKTRR	ERGFFYTPKTRR
46	CGERGFFYTPKTRRE	GERGFFYTPKTRRE	ERGFFYTPKTRRE	RGFFYTPKTRRE
47	GERGFFYTPKTRREA	ERGFFYTPKTRREA	RGFFYTPKTRREA	GFFYTPKTRREA
48	ERGFFYTPKTRREAE	RGFFYTPKTRREAE	GFFYTPKTRREAE	FFYTPKTRREAE
49	RGFFYTPKTRREAED	GFFYTPKTRREAED	FFYTPKTRREAED	FYTPKTRREAED
50	GFFYTPKTRREAEDL	FFYTPKTRREAEDL	FYTPKTRREAEDL	YTPKTRREAEDL
51	FFYTPKTRREAEDLQ	FYTPKTRREAEDLQ	YTPKTRREAEDLQ	TPKTRREAEDLQ
52	FYTPKTRREAEDLQV	YTPKTRREAEDLQV	TPKTRREAEDLQV	PKTRREAEDLQV
53	YTPKTRREAEDLQVG	TPKTRREAEDLQVG	PKTRREAEDLQVG	KTRREAEDLQVG
54	TPKTRREAEDLQVGQ	PKTRREAEDLQVGQ	KTRREAEDLQVGQ	TRREAEDLQVGQ
55	PKTRREAEDLQVGQV	KTRREAEDLQVGQV	TRREAEDLQVGQV	RREAEDLQVGQV
56	KTRREAEDLQVGQVE	TRREAEDLQVGQVE	RREAEDLQVGQVE	REAEDLQVGQVE
57	TRREAEDLQVGQVEL	RREAEDLQVGQVEL	REAEDLQVGQVEL	EAEDLQVGQVEL
58	RREAEDLQVGQVELG	REAEDLQVGQVELG	EAEDLQVGQVELG	AEDLQVGQVELG
59	REAEDLQVGQVELGG	EAEDLQVGQVELGG	AEDLQVGQVELGG	EDLQVGQVELGG
60	EAEDLQVGQVELGGG	AEDLQVGQVELGGG	EDLQVGQVELGGG	DLQVGQVELGGG
61	AEDLQVGQVELGGGP	EDLQVGQVELGGGP	DLQVGQVELGGGP	LQVGQVELGGGP
62	EDLQVGQVELGGGPG	DLQVGQVELGGGPG	LQVGQVELGGGPG	QVGQVELGGGPG
63	DLQVGQVELGGGPGA	LQVGQVELGGGPGA	QVGQVELGGGPGA	VGQVELGGGPGA
64	LQVGQVELGGGPGAG	QVGQVELGGGPGAG	VGQVELGGGPGAG	GQVELGGGPGAG
65	QVGQVELGGGPGAGS	VGQVELGGGPGAGS	GQVELGGGPGAGS	QVELGGGPGAGS
66	VGQVELGGGPGAGSL	GQVELGGGPGAGSL	QVELGGGPGAGSL	VELGGGPGAGSL
67	GQVELGGGPGAGSLQ	QVELGGGPGAGSLQ	VELGGGPGAGSLQ	ELGGGPGAGSLQ
68	QVELGGGPGAGSLQP	VELGGGPGAGSLQP	ELGGGPGAGSLQP	LGGGPGAGSLQP
69	VELGGGPGAGSLQPL	ELGGGPGAGSLQPL	LGGGPGAGSLQPL	GGGPGAGSLQPL
70	ELGGGPGAGSLQPLA	LGGGPGAGSLQPLA	GGGPGAGSLQPLA	GGPGAGSLQPLA
71	LGGGPGAGSLQPLAL	GGGPGAGSLQPLAL	GGPGAGSLQPLAL	GPGAGSLQPLAL
72	GGGPGAGSLQPLALE	GGPGAGSLQPLALE	GPGAGSLQPLALE	PGAGSLQPLALE
73	GGPGAGSLQPLALEG	GPGAGSLQPLALEG	PGAGSLQPLALEG	GAGSLQPLALEG
74	GPGAGSLQPLALEGS	PGAGSLQPLALEGS	GAGSLQPLALEGS	AGSLQPLALEGS
75	PGAGSLQPLALEGSL	GAGSLQPLALEGSL	AGSLQPLALEGSL	GSLQPLALEGSL
76	GAGSLQPLALEGSLQ	AGSLQPLALEGSLQ	GSLQPLALEGSLQ	SLQPLALEGSLQ
77	AGSLQPLALEGSLQK	GSLQPLALEGSLQK	SLQPLALEGSLQK	LQPLALEGSLQK
78	GSLQPLALEGSLQKR	SLQPLALEGSLQKR	LQPLALEGSLQKR	QPLALEGSLQKR
79	SLQPLALEGSLQKRG	LQPLALEGSLQKRG	QPLALEGSLQKRG	PLALEGSLQKRG
80	LQPLALEGSLQKRGI	QPLALEGSLQKRGI	PLALEGSLQKRGI	LALEGSLQKRGI
81	QPLALEGSLQKRGIV	PLALEGSLQKRGIV	LALEGSLQKRGIV	ALEGSLQKRGIV
82	PLALEGSLQKRGIVE	LALEGSLQKRGIVE	ALEGSLQKRGIVE	LEGSLQKRGIVE
83	LALEGSLQKRGIVEQ	ALEGSLQKRGIVEQ	LEGSLQKRGIVEQ	EGSLQKRGIVEQ
84	ALEGSLQKRGIVEQC	LEGSLQKRGIVEQC	EGSLQKRGIVEQC	GSLQKRGIVEQC
85	LEGSLQKRGIVEQCC	EGSLQKRGIVEQCC	GSLQKRGIVEQCC	SLQKRGIVEQCC

86	EGSLQKRGIVEQCCT	GSLQKRGIVEQCCT	SLQKRGIVEQCCT	LQKRGIVEQCCT
87	GSLQKRGIVEQCCTS	SLQKRGIVEQCCTS	LQKRGIVEQCCTS	QKRGIVEQCCTS
88	SLQKRGIVEQCCTSI	LQKRGIVEQCCTSI	QKRGIVEQCCTSI	KRGIVEQCCTSI
89	LQKRGIVEQCCTSIC	QKRGIVEQCCTSIC	KRGIVEQCCTSIC	RGIVEQCCTSIC
90	QKRGIVEQCCTSICS	KRGIVEQCCTSICS	RGIVEQCCTSICS	GIVEQCCTSICS
91	KRGIVEQCCTSICSL	RGIVEQCCTSICSL	GIVEQCCTSICSL	IVEQCCTSICSL
92	RGIVEQCCTSICSLY	GIVEQCCTSICSLY	IVEQCCTSICSLY	VEQCCTSICSLY
93	GIVEQCCTSICSLYQ	IVEQCCTSICSLYQ	VEQCCTSICSLYQ	EQCCTSICSLYQ
94	IVEQCCTSICSLYQL	VEQCCTSICSLYQL	EQCCTSICSLYQL	QCCTSICSLYQL
95	VEQCCTSICSLYQLE	EQCCTSICSLYQLE	QCCTSICSLYQLE	CCTSICSLYQLE
96	EQCCTSICSLYQLEN	QCCTSICSLYQLEN	CCTSICSLYQLEN	CTSICSLYQLEN
97	QCCTSICSLYQLENY	CCTSICSLYQLENY	CTSICSLYQLENY	TSICSLYQLENY
98	CCTSICSLYQLENYC	CTSICSLYQLENYC	TSICSLYQLENYC	SICSLYQLENYC
99	CTSICSLYQLENYCN	TSICSLYQLENYCN	SICSLYQLENYCN	ICSLYQLENYCN

Supplementary Table 3: 15/16-mer peptide sequences recognized by proinsulin-reactive TCRs

Peptide Name	AA Sequence
PPI:22-36	AAAFVNQHLCGSHLV
PPI:23-37	AAFVNQHLCGSHLVE
PPI:24-38	AFVNQHLCGSHLVEA
PPI:25-39	FVNQHLCGSHLVEAL
PPI:28-42	QHLCGSHLVEALYLV
PPI:29-43	HLCGSHLVEALYLVC
PPI:30-44	LCGSHLVEALYLVCG
PPI:31-45	CGSHLVEALYLVCGE
PPI:32-46	GSHLVEALYLVCGER
PPI:33-47	SHLVEALYLVCGERG
PPI:34-48	HLVEALYLVCGERGF
PPI:35-49	LVEALYLVCGERGFF
PPI:36-50	VEALYLVCGERGFFY
PPI:37-51	EALYLVCGERGFFYT
PPI:38-52	ALYLVCGERGFFYTP
PPI:39-53	LYLVCGERGFFYTPK
PPI:40-54	YLVCGERGFFYTPKT
PPI:41-55	LVCGERGFFYTPKTR
PPI:55-69	RREAEDLQVGQVELG
PPI:56-70	REAEDLQVGQVELGG
PPI:57-71	EAEDLQVGQVELGGG
PPI:58-72	AEDLQVGQVELGGGP
PPI:59-73	EDLQVGQVELGGGPG
PPI:72-86	PGAGSLQPLALEGSL
PPI:72-87	PGAGSLQPLALEGSLQ
PPI:73-87	GAGSLQPLALEGSLQ
PPI:74-88	AGSLQPLALEGSLQK
PPI:75-89	GSLQPLALEGSLQKR
PPI:76-90	SLQPLALEGSLQKRG
PPI:77-91	LQPLALEGSLQKRGI
PPI:81-95	ALEGSLQKRGIVEQC
PPI:86-100	LQKRGIVEQCCTSIC
PPI:86-100	LQKRGIVEQCCTSIC
PPI:87-101	QKRGIVEQCCTSICS
PPI:88-102	KRGIVEQCCTSICSL
PPI:89-103	RGIVEQCCTSICSLY
PPI:92-106	VEQCCTSICSLYQLE
PPI:93-107	EQCCTSICSLYQLEN
PPI:94-108	QCCTSICSLYQLENY
PPI:95-109	CCTSICSLYQLENYC

Supplementary Table 5: Primers for DRB3 and DRB4 typing

	Primer name	Sequence	Target genes	Position	Size
Forward	DRseq-F1	CAGACGTGTGCTCTTCCGATCT-ATGGTGTGTCTGAAGCTCCCTG	DRB3, DRB4	1-289	289
Reverse	DRseq-R289	CTACACGACGCTCTTCCGATCT-GGAGGTCCTTCTGGCTGTTCC			
Forward	DRB3seq-F111	CAGACGTGTGCTCTTCCGATCT-GGAGCTGCGTAAGTCTGAGTGTC	DRB3	111-404	294
Reverse	DRB3seq-R404	CTACACGACGCTCTTCCGATCT-GTCTTTGCAGGATACACAGTCACC			
Forward	DRB4seq-F116	CAGACGTGTGCTCTTCCGATCT-AGGCTAAGTGTGAGTGTCATTTCC	DRB4	116-404	289
Reverse	DRB4seq-R404	CTACACGACGCTCTTCCGATCT-GTCTTTGAAGGATACACAGTCACCT			
Forward	DRB3seq-F301	CAGACGTGTGCTCTTCCGATCT-CGGGGCCGGGTGGACAAT	DRB3	301-590	290
Reverse	DRB3seq-R590	CTACACGACGCTCTTCCGATCT-CCACTCCGAGGAACTGTTTCT			
Forward	DRB4seq-F309	CAGACGTGTGCTCTTCCGATCT-GGTGGACACCTACTGCAGAT	DRB4	309-590	282
Reverse	DRseq-R590	CTACACGACGCTCTTCCGATCT-CCACTCCGAGGAACTGTTTCC			
Forward	DRseq-F406	CAGACGTGTGCTCTTCCGATCT-CAGCCCCTGCAGCACCAC	DRB3, DRB4	406-678	273
Reverse	DRseq-R655	CTACACGACGCTCTTCCGATCT-GCTCTGTGCAGATTCAGACCGTGC			
Forward	DRB3seq-F513	CAGACGTGTGCTCTTCCGATCT-GGTGTCCACGGGCCTGATCC	DRB3	513-801	289
Reverse	DRseq-R780	CTACACGACGCTCTTCCGATCT-TCAGCTCAGGAATCCTGTTGGC			
Forward	DRseq-F513	CAGACGTGTGCTCTTCCGATCT-GGTGTCCACAGGCCTGATCC	DRB4	513-801	289
Reverse	DRB4seq-R780	CTACACGACGCTCTTCCGATCT-TCAGCTCAAGAGTCCTGTTGGC			
